# The temporal gene expression landscape of rhabdomyolysis-induced acute kidney injury reveals the timing of complement activation

**DOI:** 10.1038/s42003-025-09449-y

**Published:** 2025-12-31

**Authors:** Anne Grunenwald, Idris Boudhabhay, Margot Revel, Victoria Poillerat, Elodie Voilin, Amine Majdi, Khalil Chaibi, Stephane Gaudry, Trent M. Woodruff, Gilles Crambert, Julien Guihaire, Mohamad Zaidan, Julie Oniszczuk, Marie Frimat, Viviane Gnemmi, Marc Aletti, Hubert Nielly, Laurent Gilardin, Lubka T. Roumenina

**Affiliations:** 1 Centre de Recherche des Cordeliers, Institut National de la Santé et de la Recherche Médicale, Sorbonne Université, Université de Paris Cité, Team Inflammation, Complement and cancer, Paris, France; 2 Department of Nephrology and Hemodialysis, CHI de Poissy-St Germain en Laye, Poissy, France; 3 University Hospital Federation (FHU) COMET, Paris, France; 4 Centre de Recherche des Cordeliers, Institut National de la Santé et de la Recherche Médicale, Sorbonne Université, Université de Paris Cité, Team Integrative Cancer Immunology, Paris, France; 5 Department of Intensive Care, AP-HP, Hôpital Avicenne, Bobigny, France; 6 Tenon Hospital, INSERM UMRS1155, Sorbonne Université CORAKID, Paris, France; 7 School of Biomedical Sciences, Faculty of Medicine, University of Queensland, St Lucia, QLD Australia; 8 Centre de Recherche des Cordeliers, Institut National de la Santé et de la Recherche Médicale, Sorbonne Université, Université de Paris Cité, Team Renal Physiology and Tubulopathies, Paris, France; 9 CNRS EMR 8228 Team Métabolism and renal physiology, Paris, France; 10 Cardiac and Vascular Surgery Department, Marie Lannelongue Hospital, Groupe Hospitalier Paris Saint-Joseph, Université Paris-Saclay, Paris, France; 11 Department of Nephrology and Renal Transplantation, Assistance Publique-Hôpitaux de Paris, Bicetre Hospital, Le Kremlin-Bicêtre, France; 12 Department of Nephrology and Renal Transplantation, Foch Hospital, Suresne, France; 13 Univ. Lille, CHU Lille, Nephrology Department, Inserm U1167 - RID-AGE, Lille, France; 14 University Lille, CNRS, Inserm, CHU Lille, UMR9020-U1277 - CANTHER - Cancer Heterogeneity Plasticity and Resistance to Therapies, Lille, France; 15 Department of Pathology, CHU Lille, Lille, France; 16 Department of Internal Medicine, Percy Military Teaching Hospital, Clamart, France; 17 Department of Internal Medicine, Bégin Military Teaching Hospital, Saint-Mandé, France; 18 Sorbonne Paris-Nord University (Paris 13), Bobigny, France; 19 Department of Internal Medicine, Jean Verdier Hospital, HUPSSD AP-HP, Bondy, France; 20 Centre de Recherche des Cordeliers, Institut National de la Santé et de la Recherche Médicale, Sorbonne Université, Université de Paris Cité, Team Immunopathology and immuno-intervention therapy, Paris, France

**Keywords:** Kidney, Complement cascade, Inflammation

## Abstract

Rhabdomyolysis-induced acute kidney injury (RIAKI) involves complement activation, but its role as a therapeutic target remains unclear. We analyze urine and kidney biopsies from RIAKI patients and use a glycerol-induced mouse model to investigate complement activation and its contribution to RIAKI. Here we show that complement fragments Ba, Bb, C5a, and sC5b-9 are elevated in the urine of patients with RIAKI, and C3 staining is detected in injured tubules, often surrounded by C5aR1-expressing myeloid cells. However, pharmacologic C5 or C5aR1 inhibition fail to prevent RIAKI in mice. A kinetic analysis reveal that complement activation occurs later in the disease course, following early tubular injury and immune cell infiltration. Initial cytoprotective responses are rapidly overwhelmed, leading to tubular damage and chemokine-driven C5aR1-expressing myeloid cells recruitment. These findings suggest that complement cascade is not an initiating factor in RIAKI and underscore the multifactorial nature of this disease.

## Abstract (web summary)

Kinetic analysis of rhabdomyolysis-induced acute kidney injury reveals early tubular injury and immune cell infiltration precede complement activation. Initial protective responses fail quickly, leading to escalating damage. Timing highlights need for early, multi-targeted therapies.

## Introduction

Acute kidney injury (AKI) is a common complication of numerous acute illnesses and is associated with increased mortality regardless of its etiology^1^. AKI may occur because of medication nephrotoxicity, sepsis, surgery - particularly those associated with ischemia-reperfusion injury (IRI) or rhabdomyolysis. Rhabdomyolysis-induced acute kidney injury (RIAKI) can result from any cause of muscle damage such as trauma, hypoxia, strenuous exercise, medication, viral infections or drug abuse^2–4^. RIAKI shares common features with other AKI associated with trauma, but also other AKI causes, such as IRI, hemodynamic changes, tubular injury and innate immune response, notably driven by inflammatory monocytes^5–7^ and complement^8–10^. Patient management in RIAKI remains primarily symptomatic due to the lack of specific treatments. A detailed understanding of individual susceptibility, the mechanisms of kidney injury and its kinetics is important for developing specific treatments and optimizing standard of care. Such insights could also help identify better renoprotective strategies for the victims of natural disasters or armed conflicts, when access to dialysis is not always available^11^. The major determinant of kidney injury after rhabdomyolysis is the release into the circulation of muscle injury-derived danger-associated molecular patterns (DAMPs), including myoglobin^12^, which causes vasoconstriction, distal nephron obstruction by casts, direct tubular toxicity^13^, oxidative stress and innate immune response, notably by inflammatory monocytes^5–7^ and complement^8,9^. In the era of complement therapeutics, a deeper understanding of where complement activation fits within the broader landscape of inflammatory and tissue injury pathways is essential to determine its potential as a viable therapeutic target.

We hypothesize that the understanding of the sequence of events in the RIAKI will allow us to predict whether a complement system inhibition could be a potential therapeutic strategy in RIAKI.

## Results

### Complement as a urine biomarker in RIAKI patients

**Fig. 1:**
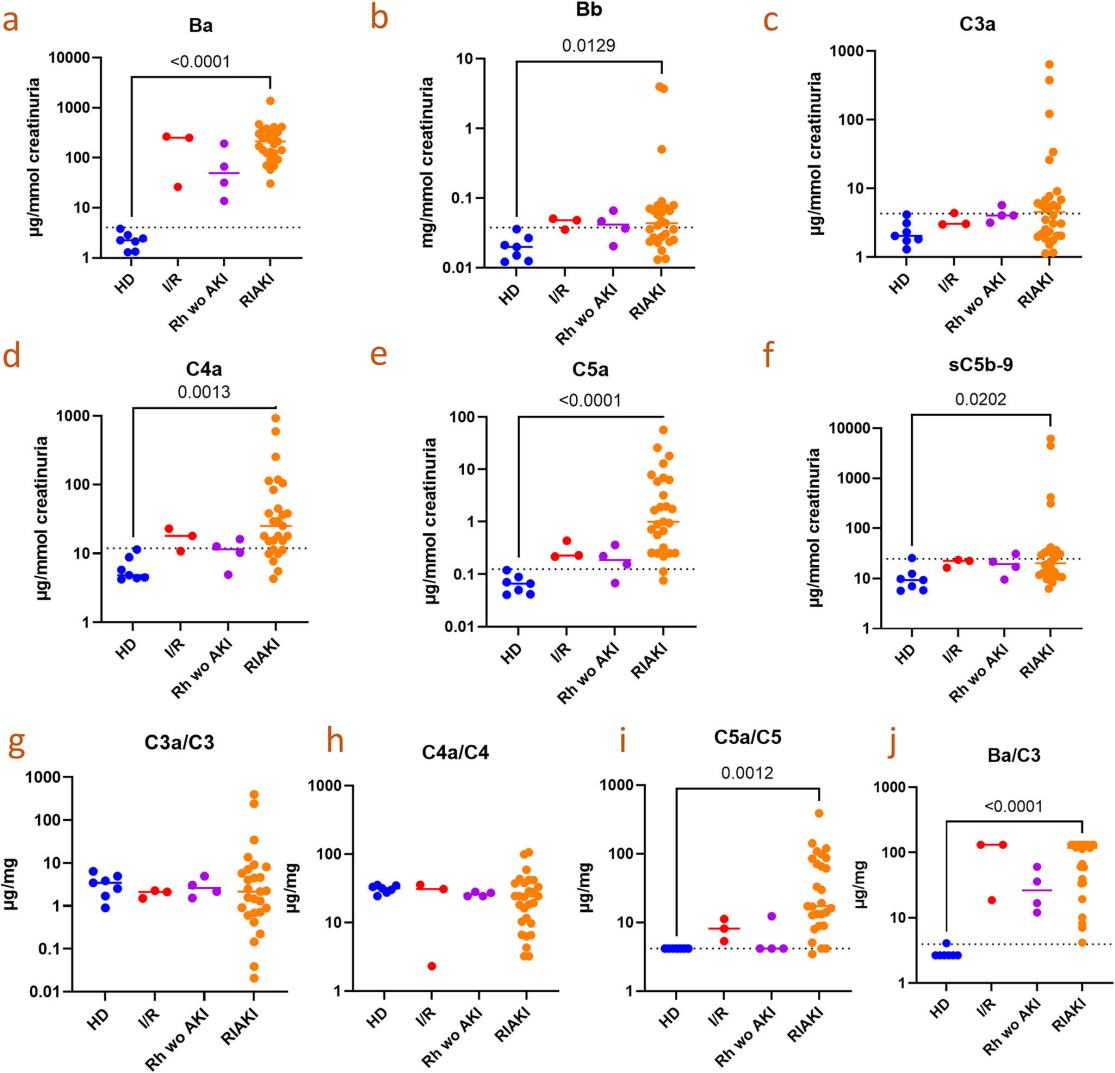
Complement activation fragments increase in the urine of patients with AKI. Ratio of complement activation fragments to creatinuria in urines of healthy donors (HD, *n* = 7), patients with renal ischemia reperfusion (IR, *n* = 3), rhabdomyolysis (Rh) without (wo) acute kidney injury (AKI), *n* = 4, or Rhabdomyolysis induced AKI (RIAKI, *n* = 27): Ba (**a**), Bb (**b**), C3a (**c**), C4a (**d**), C5a (**e**) and soluble C5b9 (sC5b9) (**f**). Ratio of complement active fragments to complement complete proteins C3a/C3 (**g**), C4a/C4 (**h**) C5a/C5 (**i**), and Ba/C3 (**j**) (*p < 0.05, **p < 0.01, ***p < 0.001, ****p < 0.0001, Kruskal–Wallis test with Dunn’s correction for multiple comparisons to HD, dotted lines represent mean of HD + 2 standard deviations).

Multiplexed evaluation of complement components and activation fragments of complement cascade (illustrated in Supplementary Fig. S1a) in biological fluids, part of the Humoral Complementomics approach^14,15^ was applied in urine. Results are presented upon urine creatinine normalization (Fig. 1, Supplementary Fig. S1). This approach revealed an increase in Ba levels in all rhabdomyolysis patients, both with and without AKI, as well as in all patients with IRI (Fig. 1a). However, in the Kruskal–Wallis test, only the RIAKI group reached statistical significance. A comparison between healthy controls and the rhabdomyolysis without AKI group or the IRI group were significant using the Mann–Whitney test. Mean creatininuria-normalized Bb (Fig. 1b), C4a (Fig. 1d), C5a (Fig. 1e), and sC5b-9 (Fig. 1f) were significantly higher in RIAKI patients, although all the RIAKI patients did not display this increase. C5a was increased in all RIAKI patients except 2, but not C3a (Fig. 1c). Mean C1q, C2, C3, C4, C5, FD, FP, FH and FI (Supplementary Fig. S1b–j) were also significantly increased in patients with RIAKI but not in rhabdomyolysis patients without AKI. No differences were observed regarding C3a/C3 (Fig. 1g), and C4a/C4 ratios (Fig. 1h). Conversely, a significant increase in the C5a/C5 ratio was noted (Fig. 1i), which appeared to have a 75% specificity to discriminate from rhabdomyolysis with or without AKI. Lastly, Ba/C3 ratio (Fig. 1j) was elevated regardless creatinine levels. The ratio for Ba was made over C3 as measurement of FB is lacking currently in the multiplex ELISA kit.

### Staining for complement proteins in proximal tubules in RIAKI patients

**Fig. 2:**
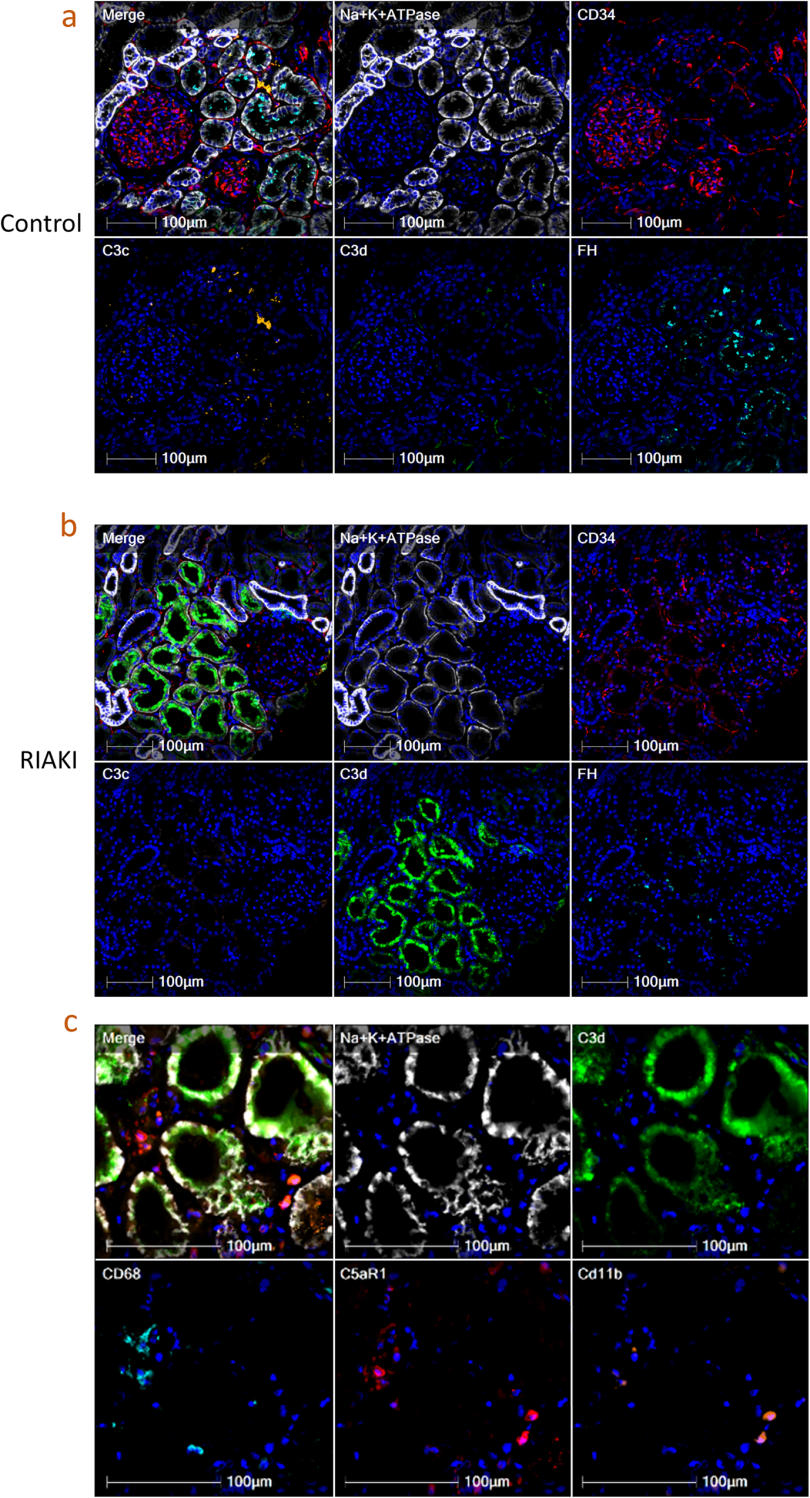
RIAKI in patients is characterized by complement staining of the proximal tubules and infiltration of the kidney with myeloid cells, having a complement-responsive phenotype. Multiplex sequential immunofluorescence staining using the in situ Complementomics approach of **a** control peritumoral kidney tissue and **b** an example of a kidney biopsy of a RIAKI patient with CD34 (red) and Na+K+ATPase (white) together with complement proteins C3c (orange), C3d (green) and Factor H (FH, cyan) in control kidney biopsies (left) and RIAKI patients (middle). **c** Multiplex sequential immunofluorescence staining of Na+K+ATPase (white), C3d (green), CD68 (cyan), C5aR1 (red) and CD11b (orange) in a RIAKI patient.

The application of multiplexed seqIF in situ Complementomics approach^16^ to five kidney biopsies of RIAKI patients (clinical data in Supplementary Table 2), in comparison to three peri-tumoral tissues of renal cancer confirmed the presence of C3 tubular staining (odds ratio = 5.3, 95% CI [5.1–5.4]) using a polyclonal anti-C3d antibody. We detected presence of more FB (odds ratio = 3.1, 95% CI [3–3.3]) and less FH staining in the RIAKI tubules (odds ratio = 0.28, 95% CI [0.27–0.29]) (Fig. 2a–c)^17^. Notably, C3d-positive tubules were frequently surrounded by macrophages (CD68+) expressing C5aR1 and CD11b (Fig. 2c). C3c staining was also detected in some patients but it is rarer, as the biopsies are often taken at distance from the onset of AKI. C3c is a marker of ongoing complement activation, while C3d, more stable to proteolytic cleavage, remains at long term after activation.

### Immune infiltrate in RIAKI has a complement-responsive phenotype

**Fig. 3:**
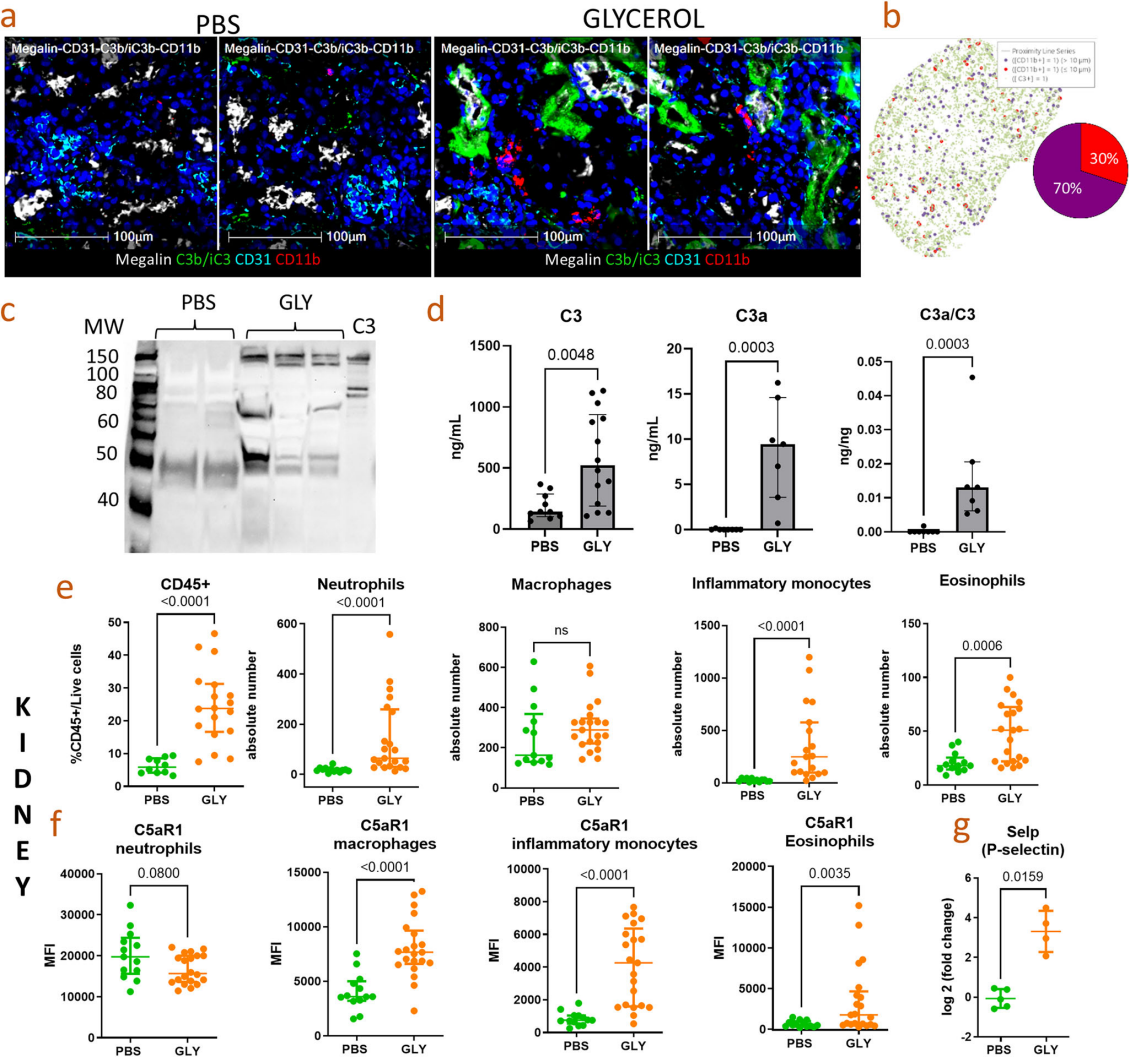
Complement-responsive phenotype of the myeloid infiltrate in RIAKI. Multiplexed sequential immunofluorescence (SeqIF) staining for: **a** C3b/iC3b (green), CD31 (cyan, endothelial cells), Megalin (white, proximal tubular cells) and CD11b (red, immune cells) on snap frozen kidneys of C57Bl6 WT mice 24 h after injection by PBS (left) or Glycerol (right), two representative images of each. **b** Spatial plot of proximity analyzes between C3b/iC3b positive tubules (green) and CD11b positive cells (red, immune cells). Presence of C3 and its activation fragments in the urine of glycerol-injected mice, evaluated (**c**) by western blot and (**d**) by ELISA, measuring C3, C3a and the C3a/C3 ratio for WT-PBS and WT-GLY mice at 24 h after injection of PBS or Glycerol. **e** Evaluation by flow cytometry of the immune infiltrate on fresh whole kidneys 24 h after glycerol injection, regarding CD45+ cells (% of Live/Dead negative cells) (left), Neutrophils (LD−/CD45+/CD11b+/CD11c−/SSC high/LY6G+) (middle left), macrophages (LD−/CD45+/CD11b+/CD11c−/LY6G−/Ly6C low) (middle), inflammatory monocytes (LD−/CD45+/CD11b+CD11c−/SSC low/LY6G−/Ly6C high) (middle right), eosinophils (LD−/CD45+/CD11b+/CD11c−/SSC high/LY6G−, right). The presented results are from 3 independent experiments with total of 14 PBS and 22 GLY injected mice. **f** Evaluation by flow cytometry of the Mean Fluorescence Intensity (MFI) of C5aR1 on neutrophils (left), macrophages (middle left), inflammatory monocytes (middle right), eosinophils (right). Presented results are from 3 independent experiments with total of 14 PBS and 22 GLY injected mice. **g** P-selectin expression measured by Quantigene. *p < 0.05, **p < 0.01, ***p < 0.001, ****p < 0.0001, Mann–Whitney test.

We explored complement deposits and immune infiltration in a mouse model of RIAKI. We confirmed the muscle injury in the glycerol-injected mice by Creatin Kinase (CK, *p* = 0.0042) and Aspartate-Amino-Transferase (ASAT, *p* < 0.0001) (Supplementary Fig. S2a) and the kidney injury assessed by urea (*p* < 0.0001) and creatinine (*p* < 0.0001) (Supplementary Fig. S2b) at 24 h post-treatment. Application of multiplexed seqIF to kidneys of glycerol-injected mice revealed a pattern of C3b/iC3b staining localized to proximal tubules positive for Megalin but absent in glomeruli, as indicated by CD31 staining (Fig. 3a). Spatial analysis showed that 30% of CD11b-positive myeloid cells were within 10μm of C3b/iC3b-positive tubules, suggesting a potential interaction (Fig. 3a, b). Moreover, C3 activation was detected in the urine of the RIAKI mice at H24 post injury by western blot (Fig. 3c) and ELISA (*p* = 0.0048) (Fig. 3d). C3a (*p* = 0.0003) and the C3a/C3 ratio (*p* = 0.0003) were also elevated in the urine of the RIAKI mice (Fig. 3d).

Inflammatory monocytes/macrophages are considered key drivers of RIAKI^5–7,18^. To determine if infiltrating immune cells expressed increased expression of C5aR1, we explored the immune infiltrate at 24 h in the glycerol-injected mice. Flow cytometry analysis of fresh whole kidney tissue at 24 h, confirmed the significant increase of CD45+ cells among live cells (*p* < 0.0001). This immune infiltrate consisted of neutrophils (p < 0.0001), inflammatory monocytes (*p* < 0.0001) and eosinophils (*p* = 0.0006), but not resident macrophages (Fig. 3e). C5aR1 was increased in resident macrophages (*p* < 0.0001), inflammatory monocytes (*p* < 0.0001), and eosinophils (*p* = 0.0035), but not in neutrophils, in which there was a trend towards a decreased expression (*p* = 0.08, activation marker). However, the absolute level of C5aR1 expression was the highest in neutrophils (Fig. 3f). With an increase of P-selectin (Fig. 3g), the diapedesis of granulocytes, monocytes, eosinophils can be facilitated and indeed their blood concentration is increased (Supplementary Fig. S3).

### Despite a complement-responsive myeloid infiltrate, targeting of C5/C5aR1 axis did not prevent RIAKI

**Fig. 4:**
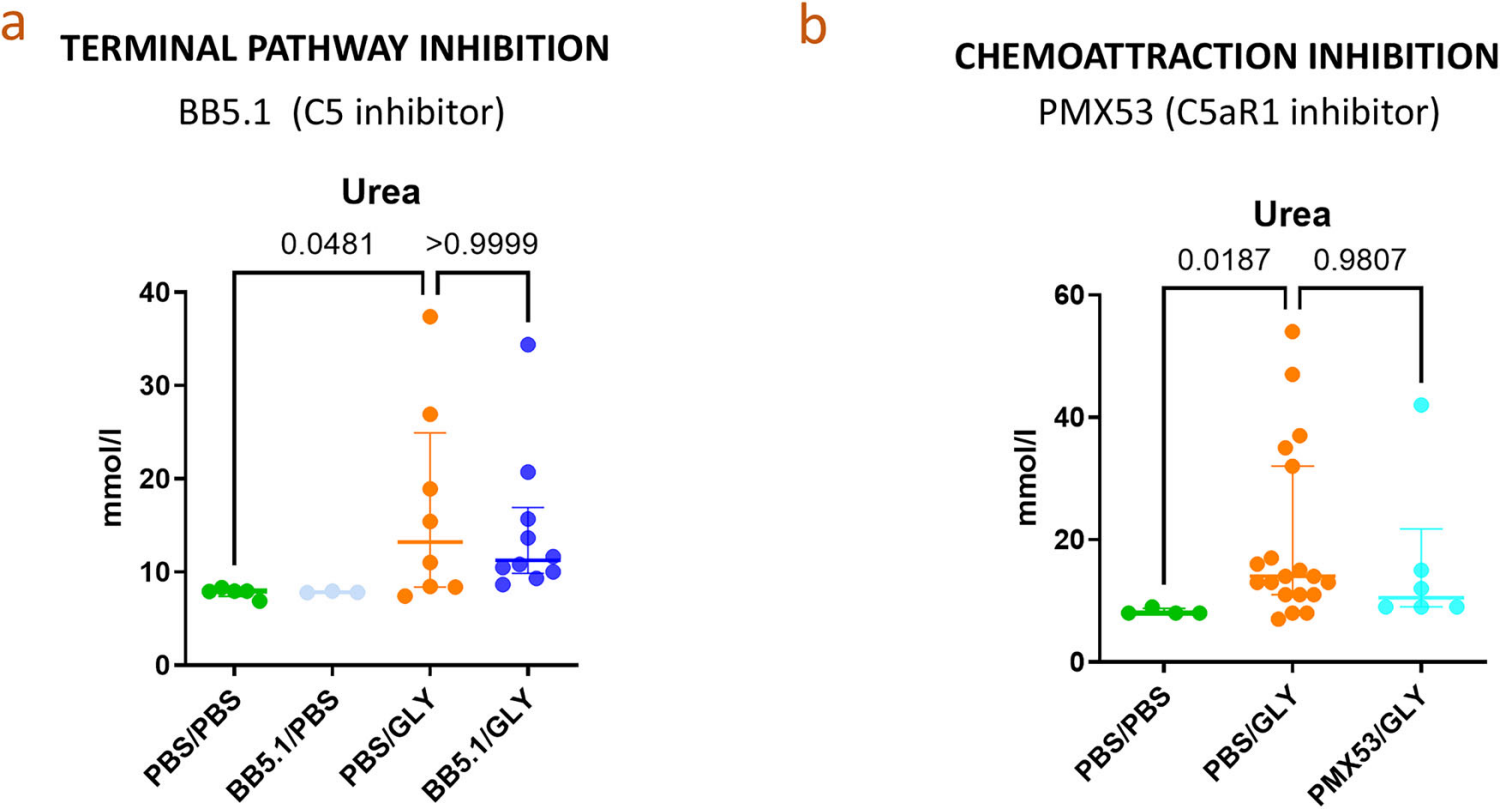
Despite a complement-responsive myeloid infiltrate, the targeting of C5/C5aR1 axis does not prevent AKI. **a** C5 inhibition does not prevent kidney injury. BUN of WT mice injected by glycerol, preventively treated by C5 inhibitor BB5.1. The presented results are from one experiment with 5 PBS/PBS,3 BB5.1/PBS, 10 PBS/GLY, 10 BB5.1/GLY injected mice. **b** C5aR1 inhibition does not prevent kidney injury. BUN of C57Bl6 WT mice injected by glycerol, preventively treated by C5aR1 inhibitor PMX53. The presented results are from one experiment (out of 2 performed with two different sources of PMX53) with 4 PBS, 19 GLY and 6 - preventively treated by PMX53 - GLY injected mice. (*p < 0.05, Kruskal–Wallis test with Dunn’s correction for multiple pairwise comparisons).

As the immune infiltrate was C5a-responsive, we inhibited preventively C5 in glycerol-injected mice with a murine equivalent of Eculizumab - BB5.1, or C5aR1 with PMX53. Neither BB5.1 nor PMX53 prevented muscle injury (Supplementary Fig. S4a, b) or the urea elevation in plasma (Fig. 4a, b).

### Kinetic analysis of complement in RIAKI mice

To understand this lack of protection, we analyzed plasma and renal markers at different end points in a kinetic model of RIAKI (Supplementary Fig. S5a). Muscle injury was evidenced by an increase in the plasma ASAT, already detectable at 3 h, followed by a significant rise of plasma urea and creatinine from 12 h onward (Supplementary Fig. S5b). Our model involved kidney but not liver injury, even though in trauma patients both can co-occur, increasing severity^19^.

**Fig. 5:**
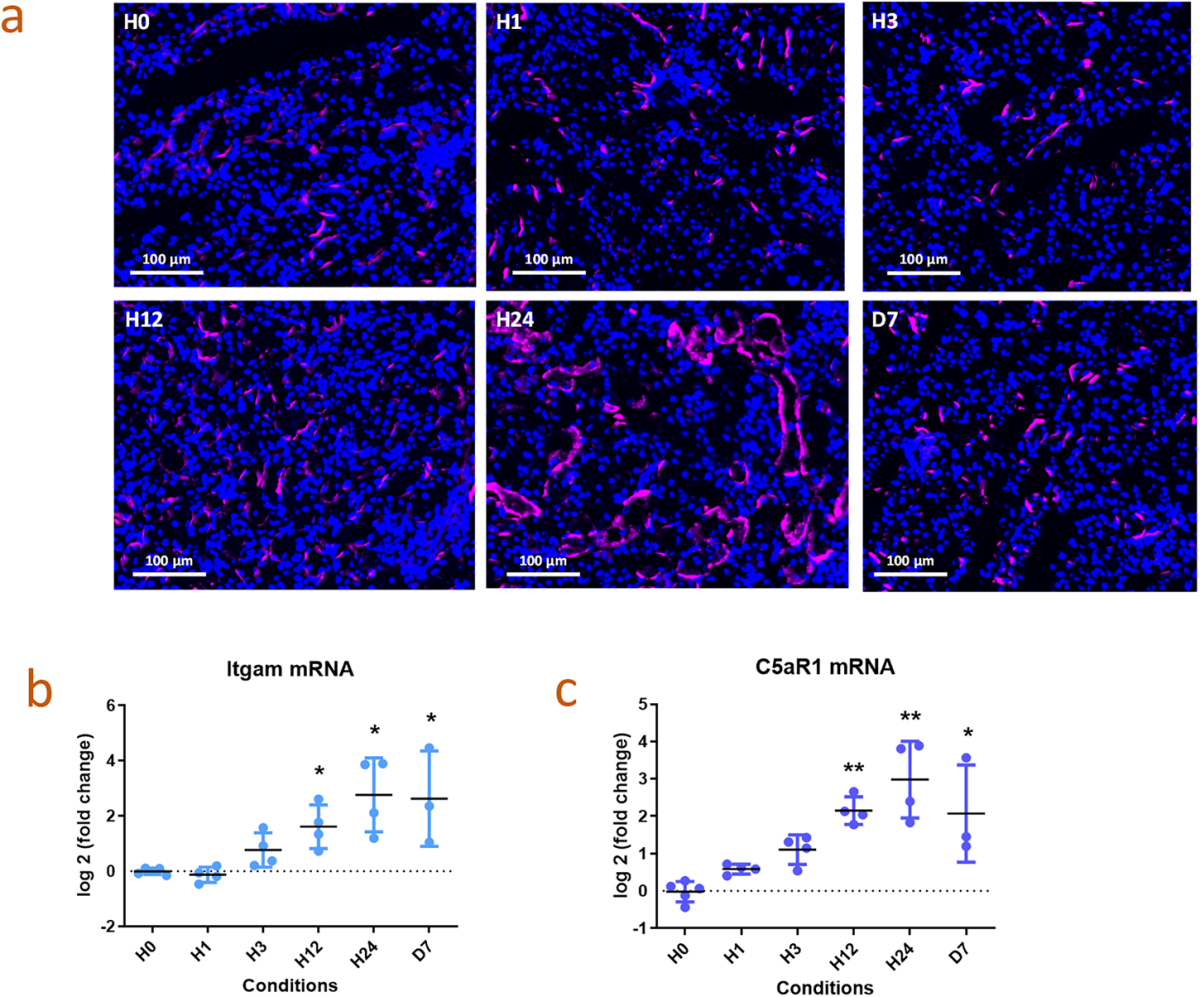
Kinetic of appearance of C3 staining, CD11b and C5AR1 gene expression. **a** Representative images of C3b (pink) immunofluorescence on snap-frozen mouse kidneys 0, 1, 3, 12, 24 h and 7 days after injection by glycerol, original magnification 20. **b** Differential expression of Itgam (CD11b, component of the complement Receptor 3) and **c** C5aR1 (C5a Receptor 1) by QuantiGene in snap-frozen kidney of C57Bl6 WT mice at 0, 1, 3, 12, 24 h and 7 days after injection by glycerol. Presented results are from one experiment with 5 mice at time 0, 4 mice at 1 h, 3 h, 12 hours and 24 h each, and 3 mice at day 7.

We studied the kinetic of C3b/iC3b staining in the kidneys and observed that it appears rather late, at about 24 h, after immune cell infiltration (Fig. 5a). We observed an increase in complement receptors CD11b (*Itgam*) and C5aR1 gene expression assessed by Quantigene, likely reflecting immune cells infiltration (Fig. 5b, c), which was maximal at 24 h. The timing of Itgam and C5aR1 overexpression, which is similar to the one of IRI, as demonstrated by online single cell dataset (https://humphreyslab.com/), could be attributed to the increase of different myeloid populations in RIAKI (Supplementary Fig. S6a, b)^20,21^.

### Following an early anti-inflammatory and antioxidant state, RIAKI kidneys display strong tubular injury markers from H12

**Fig. 6:**
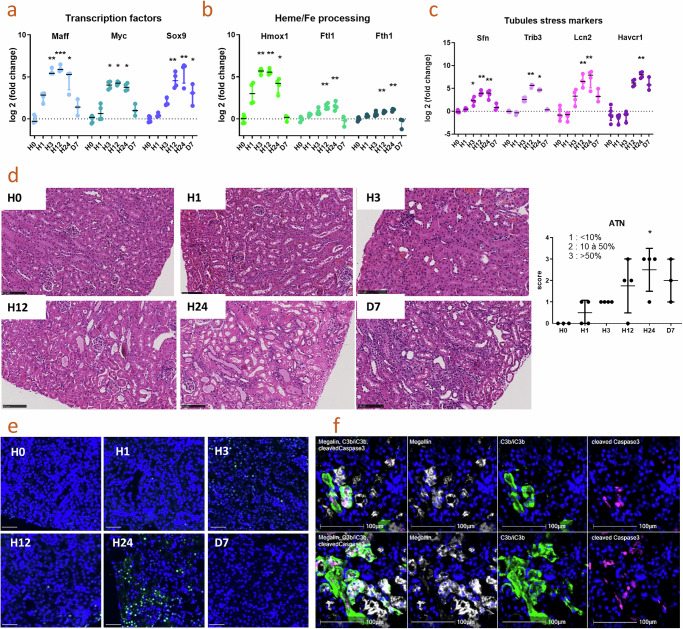
Kinetic of tubular injury of the RIAKI mouse model. Kinetic studies were performed on snap-frozen kidneys at 0, 1, 3, 12, 24 hours (*n* = 5/time point) and 7 days (*n* = 3) after injection by glycerol. **a** Cytoprotective transcription factors Maff, Myc and Sox9 and **b** anti-oxidant genes Hmox1 (Heme oxygenase 1), Ftl1 (ferritin light chain) and Fth1 (ferritin heavy chain). **c** Upregulation of classical tubular injury markers Havcr1 (Kim-1) and Lcn2 (NGAL) as well as Sfn (Stratifin) and Trib3 (Tribbles Pseudokinase 3), evaluated by QuantiGene. **d** Hematoxylin/eosin staining on paraffin-embedded kidneys (scale bar 100 µm) and evaluation of acute tubular necrosis (% of injured tubules on the total number of tubules, *p* = 0.0149). **e** TUNEL staining. Scale bar 50 µm. *p < 0.05, **p < 0.01, ***p < 0,001; Kruskal–Wallis test with Dunn’s correction for multiple pairwise comparisons. **f** Two representative sections of frozen kidneys from GLY mice showing proximal tubules (Megalin positive, white) positive for C3b/iC3b (green) and cleaved Caspase 3 (purple) 24 h after glycerol injection. Presented results are from one experiment.

To elucidate other mechanisms which could be implicated in renal injury during RIAKI, we applied the gene set of the RIAKI transcriptomic signature to our kinetic model on whole kidney sections. The first to be upregulated were transcription factors MafF (*Maff*), *Myc* and *Sox9* (Fig. 6a) as well as Heme oxygenase -1 (*Hmox1*), ferritin light (*Ftl*) and heavy (*Fth*) chain (Fig. 6b). They were significantly upregulated at H3 (and even starting at H1 for *Maff* and *Hmox1* without reaching significance). These proteins are involved in the cytoprotection and detoxification of heme and iron. They were followed by a change in the expression of tubular injury markers such as Neutrophil gelatinase-associated lipocalin (NGAL, *Lcn2*) and other genes, hallmarks of tubular aggression in other models of AKI, notably ischemic AKI (*Sfn, Trib3*) (Fig. 6c). Other tubular genes, derived from the RIAKI transcriptomic signature were downregulated during the course of renal injury, such as proximal tubules markers X-Prolyl Aminopeptidase 2 (*Xnpep2*), Sodium-dependent phosphate transport protein 2C (*Slc34e3*), C-type lectin domain family 2 member H (*Clec2h*), Cytochrome P450 2d26 (*Cyp2d26*), Meprin A Subunit Beta (*Mep1b*) (Supplementary Fig. S7a), and distal tubules markers Na+/K+/2Cl- cotransporter (NKCC2, *Slc12a1*) and Transmembrane Protein 207 (*Tmem207*) (Supplementary Fig. S7b). In agreement with this tubular signature, the severity of acute tubular necrosis (ATN), semi-quantified on hematoxylin-eosin staining, increased progressively and reached its maximum at H24 (Fig. 6d). This was associated with higher apoptosis, as illustrated by the TUNEL staining (increased from H12) (Fig. 6e), and the cleaved caspase-3 positive staining in megalin+ proximal tubules at H24 (Fig. 6f).

### RIAKI mice express fibrosis/repair patterns, which are not fully resolved at 7 days post injury

No change was evidenced for T cells apart from the latest time point at 7 days (Supplementary Fig. S8a left, middle), like fibroblasts (Supplementary Fig. S8a right) suggesting that T cells may be more implicated in the fibrosis/repair process during RIAKI. Concomitantly, several genes involved in both repair and fibrosis were upregulated often from H12 onwards, such as Collagens, type I, alpha 1 (*Col1a1*), *Col1a3*, Fibronectin 1 (*Fn1*), fibrinogen gamma chain (*Fgg*) and Transforming Growth Factor beta (*Tgfb1*), thus suggesting an early impact on late lesions (Supplementary Fig. S8b).

### This tubular injury is associated with overexpression of immune cell-attracting chemokines

**Fig. 7:**
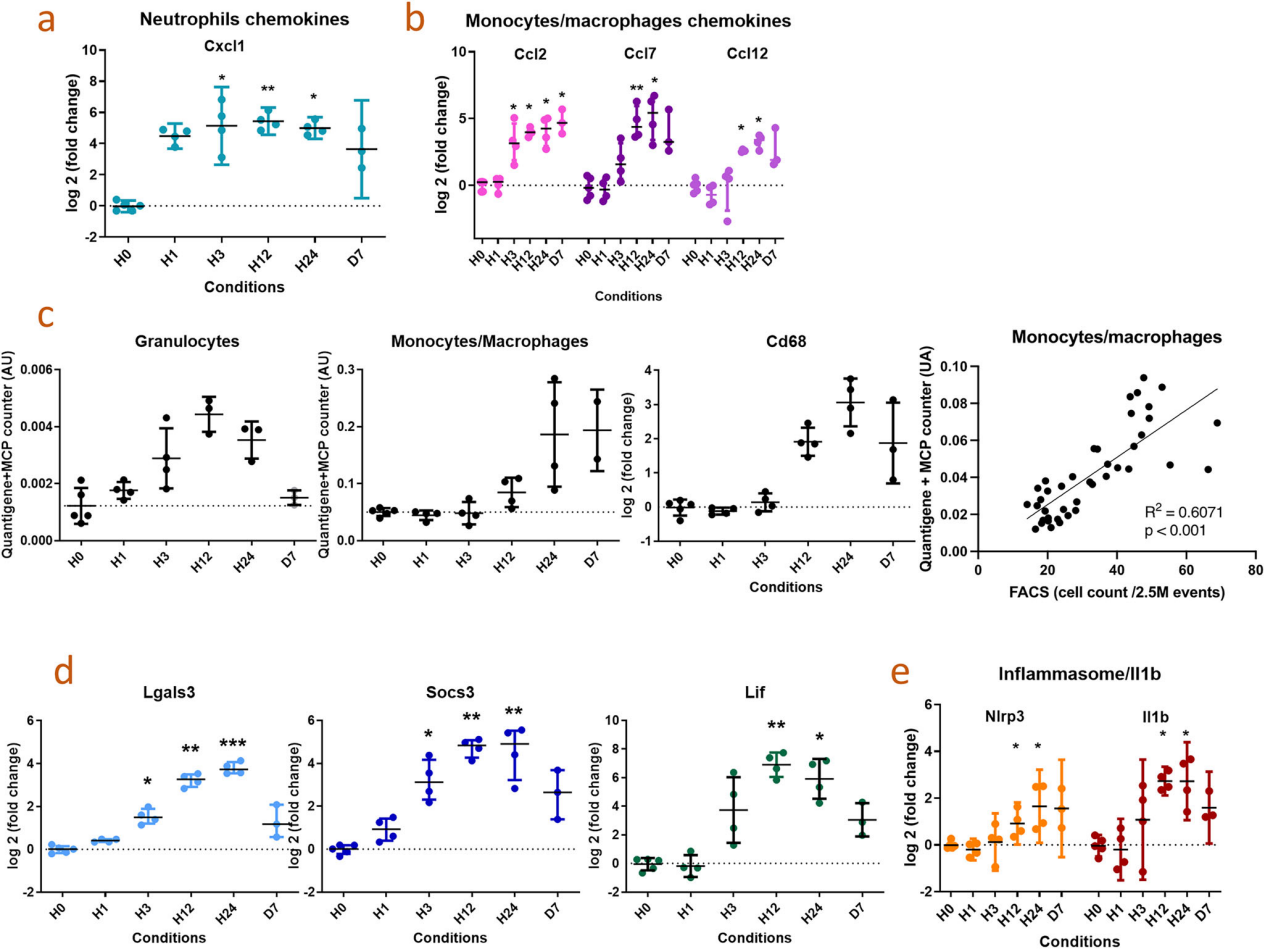
Kinetic of the inflammatory markers in the RIAKI mouse model. Differential expression of **a** Neutrophils - Cxcl1 (CXC motif chemokine ligand 1) and **b** inflammatory monocytes/macrophages - Ccl2 (CC motif chemokine ligand 2), Ccl7, Ccl12. **c** Estimation of relative abundancy of granulocytes and monocytes/macrophages (Mo/Mac) from normalized QuantiGene median values of genes analyzed with mMCP counter tool (left, middle). Correlation between the mMCP counter tool estimation for Mo/Mac from one of the kidneys, at 24 h (peak of inflammatory mediators), correlated with the flow cytometry evaluation of the inflammatory monocytes infiltrate measured in the contralateral kidney of the same mice (right). Differential regulation of inflammatory genes **d** Lgals3 (galectin 3, left), Socs3 (Suppressor of cytokine signaling 3, middle), Lif (Leukemia inhibitory factor, right) and **e** inflammatory genes related to the inflammasome formation Nlrp3 (NLR Family Pyrin Domain Containing 3) and IL1b (Interleukin 1 beta) (right). Gene expression was evaluated by Quantigene on kidney at 1, 3, 12, 24 h and 7 days after injection by glycerol. Different time points were compared to control condition (H0). *p < 0.05, **p < 0.01, ***p < 0,001; Kruskal–Wallis test with Dunn’s correction for multiple pairwise comparisons. Presented results are from one experiment with 5 mice at time 0, 4 mice at 1 h, 3 h, 12 h and 24 h each, and 3 mice at day 7.

Starting at H3, we detected an upregulation of *Cxcl1*, followed by *Ccl2*, *Ccl7* and *Ccl12* from H3 to H24 (Fig. 7a, b). Using the murine Microenvironment Cell Population (mMCP)-counter tool, we observed an increase in the granulocytes and monocytes/macrophages populations in the hours following the increase of the respective attracting cytokines (Fig. 7c left, middle). The increase in macrophages was confirmed by the expression of *Cd68*, a macrophage marker which is not in the mMCP-counter panel (Fig. 7c middle right). However, due to the heterogeneity of the mouse model, the results of the MCP counter did not reach statistical significance, even though they illustrate the kinetics of immune infiltration. The estimated relative abundancy of the monocytes/macrophages by mMCP correlated with the measurements obtained by flow cytometry (Fig. 7c right). Other inflammatory mediators such as *Lgals* (galectin 3), *Socs3*, *Lif* (Fig. 7d), and inflammasome-related genes including *Il1b* and *Nlrp3* (Fig. 7e), were also significantly upregulated in the H12-H24h period.

## Discussion

This study provides a comprehensive kinetic analysis of RIAKI, identifying key temporal events in injury initiation, immune response, and partial recovery (Supplementary Fig. S9). Complement activation fragments, especially C5a, were found in patients urine and C5aR1-expressing myeloid cells surrounded C3-positive tubules both in human and murine RIAKI. Despite this complement activation and the presence of urinary biomarkers, pharmacologic inhibition of the C5/C5aR1 axis failed to protect against kidney injury. Our kinetic analysis revealed that kidneys initially mount a protective anti-inflammatory and antioxidant response, but this phase is rapidly overtaken by tubular injury, immune infiltration, and complement activation, underscoring the multifactorial nature of RIAKI and the need for broader therapeutic strategies.

Urinary complement fragments correlate more closely with renal pathology than plasma measurements^22–30^, and may thus serve as early predictors of kidney damage or progression to chronic disease. We found Ba, Bb, C5a, and sC5b-9 in the urine of RIAKI patients with C5a and C5a/C5 ratio distinguishing AKI from uncomplicated rhabdomyolysis. Intact complement proteins were also elevated in the urine. This likely reflects a combination of passive leak of plasma proteins but also local synthesis of complement in the tubulointerstitial space, similarly to IRI^22^. The significant elevation of the activation fragment over intact protein ratios indicates enhanced cleavage relative to the amount of intact protein, suggesting true complement activation rather than mere leakage. Even if C3a/C3 ratio lost significance, detection of C3a still implies enzymatic processing. Generation of C3a in the tubular compartment could affect local inflammation and contribute to tissue injury. Importantly, Ba was also elevated in patients without overt AKI, suggesting subclinical tubular injury. Levels of Ba may also discriminates between patients who have AKI recovery and patients who develop persistent AKI^31^. Complement activation was also evident at the tissue level. In kidney biopsies from RIAKI patients, we observed prominent C3 staining in proximal tubules, accompanied by increased factor B and decreased factor H. These tubules were frequently surrounded by CD68+ macrophages expressing C5aR1 and CD11b. The glycerol-induced RIAKI mouse model reproduced key human findings, including elevated urinary complement fragments and C3 staining in proximal tubules and similar spatial arrangements of C5aR1+ macrophages. Moreover, infiltrating CD11b+ myeloid cells, particularly inflammatory monocytes and eosinophils, overexpressed C5aR1. Therefore, complement fragments not only reflect injury but may also influence immune cell localization and activation within the tubulo-interstitium. This complement-responsive infiltrate aligns with previous studies showing a central role for monocytes in RIAKI pathology^5–7,18^.

The presence of complement activation products in the urine of RIAKI patients found here, the protective effect of congenital C3 deficiency in mice^8^, the overexpression of C5aR1 on the kidney-infiltrating myeloid cells and the partial protective effect of both complement consumption by CVF in rats^9^ and hepatic FH deficiency in mice^8^, together logically suggest that complement cascade activation is a pathogenic driver of RIAKI. These data support the hypothesis that complement inactivating agents could have a therapeutic effect. Despite the clear evidence of complement activation, neither C5 inhibition with BB5.1 nor C5aR1 blockade with PMX53 mitigated renal injury. Contrary to anti-C5, an antibody that may not cross the glomerular barrier, C5aR1 blocker PMX53 is a small molecule which is likely to access the tubulo-interstitial space, suggesting that the lack of efficacy is not due to inadequate drug delivery. These findings mirror similar challenges in IRI^7–41^, where therapeutic inhibition has had limited success, despite some C1-inhibitor effect against GFR decrease^32,33^. This suggest that complement activation in RIAKI is likely an amplifying rather than initiating event.

Analysis of the kinetics of injury supports this interpretation. Complement activation, marked by renal C3 staining and transcriptional upregulation of C5aR1 and Itgam, followed the peak of tubular injury and inflammatory markers. These temporal dynamics, comparable to those observed in IRI models^20,21^, indicate that complement acts downstream of initial insult and immune cell recruitment.

In the first hours post-injury, the kidneys mounted an anti-inflammatory and antioxidant response, with increased expression of transcription factors such as Maff, which can heterodimerizes with Bach1 to bind Maff recognition element (MARE)^34–36^, present in the promoters of heme and iron detoxifiers^37^ heme oxygenase-1 and ferritin. Notably, heme oxygenase-1 was among the earliest and most robustly induced genes. It is the master heme-degrading enzyme and a primary cytoprotective effector in RIAKI^38,39^. In addition to its antioxidant and iron-sequestering functions, HO-1 activity also results in dampening of inflammation and complement activation^37^, reinforcing its central role in the defense against RIAKI. In addition, Myc^40^ and Sox9^41^ transcription factors, known for their anti-inflammatory and antioxidant properties, contribute to tubular repair. However, by 12 h post-injury, these protective mechanisms were overwhelmed, as evidenced by the upregulation of tubular injury markers like NGAL (Lcn2), Sfn, and Trib3, and the appearance of acute tubular necrosis. Ferroptosis and oxidative stress are likely contributors to this transition^42^.

Heme and oxidative stress also triggered the inflammasome and pro-inflammatory and pro-fibrotic galectin 3 as previously described^43–46^. Moreover, injured tubules started to express chemoattractants, such as Cxcl1 for neutrophils and Ccl2, Ccl7 and Ccl12 for inflammatory monocytes. This resulted in increased systemic circulation of granulocytes and inflammatory monocytes, while endothelial P-selectin likely facilitated their diapedesis into the injured kidney. The timing of these events suggests that innate immune activation amplifies pre-existing tubular injury rather than initiating it. The chemokines secreted by the injured tubules attract the granulocytes and inflammatory monocytes to the kidney even before the complement activation.

Seven days post-injury, signs of unresolved inflammation and (mal)adaptive repair/fibrotic remodeling persisted. While most immune cell populations declined, there was an increase in T cells and fibroblasts, along with continued expression of fibrosis-related genes including Col1a1, Fn1, Fgg, and Tgfb1. This transition toward maladaptive repair is consistent with known trajectories from AKI to chronic kidney disease^47,48^.

Our findings must be interpreted in light of certain limitations. Due to practical constraints, the number of mice per time point in our kinetic model was limited, reducing statistical power for some analyses. Furthermore, extended longitudinal follow-up would be necessary to evaluate progression toward irreversible fibrotic remodeling. The number of patient biopsies studied here is also limited, due to the rarity of these tissues. Moreover, matched urine samples from these patients were not available as the biopsies were collected retrospectively. Nevertheless, the concordance we observed between human and mouse data here suggest possible generalization of the mouse finding to human disease.

Our data highlight the key role of HO-1 induction and iron sequestration to limit oxidative stress early in RIAKI, which could be a step for therapeutic intervention by HO-1 overexpression or myoglobin or heme scavenging^5,8,37,39,49^. Moreover, early rise in chemokines and inflammatory monocyte recruitment and their downstream effectors may be targeted to prevent immune-mediated kidney injury^5,50^. Further studies are needed to identify optimal therapies, or combinations thereof, that target both heme-driven oxidative stress and inflammation in patients, as obvious candidates from mouse knockout studies, such as terminal complement cascade inhibition here and NLRP3 inflammasome blockade^51^, did not meet expectations.

In summary, RIAKI is initiated by muscle-derived myoglobin toxicity and progresses through a complex sequence of oxidative stress, immune cell recruitment, and complement activation. While complement plays a role in modulating the inflammatory response, our data suggest that targeting the C5/C5aR1 axis alone is insufficient. Effective therapeutic approaches will likely need to address multiple components of injury, including heme toxicity, oxidative stress, and immune activation, to achieve renoprotection in this setting.

## Materials and methods

### Collection of patients’ urine and biopsies

Urine of 32 patients with rhabdomyolysis (with a CPK cut-off > 1000IU/L) with (*n* = 28) or without (*n* = 4) renal failure, described in Supplementary Table 1; were collected from the AKIKI2 Biobank, Begin Military Teaching Hospital, Percy Military Teaching Hospital, Jean Verdier Universitary Hospital, Lille University Hospital, and European George Pompidou hospital. These urines were compared to those of 7 healthy donors and 3 post-cardiac surgery patients with extracorporeal circulation at 24 h (Marie Lannelongue Hospital) as examples of ischemia/reperfusion injury (IRI). All procedures were performed according to national ethical guidelines (agreement obtained in the framework of the study AKIKI 2, NCT03396757. The study protocol and information forms were approved by the competent French legal authority (Comité de Protection des Personnes Sud Est V, 7 February 2018). Urine samples were aliquoted and frozen rapidly after sampling, at −20 °C until transfer for long term storage at −80 °C. Kidney biopsy from 5 patients with RIAKI and 3 kidney peritumoral tissues were retrieved (clinical data in Supplementary Table 2). All patients gave informed consent for the use of their plasma or part of the biopsy for scientific purposes. All procedures were performed according to national ethical guidelines and were in accordance with the Declaration of Helsinki. All ethical regulations relevant to human research participants were followed.

### Complement quantification in urine from patients, using Multiplex ELISA

Concentrations of C1q, C2, C3, C4, C5, FD, FP (QuidelOrtho 7 plex kit HQ2M210316 16400 03) and Ba, C3a, C5a, Bb, sC5b-9, FI, FH, C4a (QuidelOrtho 8 plex kit HQ1M210421 16400 01) were assessed by multiplex ELISA^14,15^ on urine samples diluted at 1/100. Creatinuria was measured using Konelab Chemistry Analyzer equipment (ThermoFisher Scientific). Urine samples were thawed on ice. The protocols of the manufacturer have been strictly followed. Specific fluorescence signal for each protein was detected by Q-View Imager LS and results were analyzed using Q-View™ Software as proposed by the Quidel company. As Ba levels exceeded the scale on the multiplex assay, Ba levels were assessed independently in a monoplex Elisa (Ba fragment EIA MicroVue, QuidelOrtho). Of note, C3a levels are often too low to be detected with the 1/100 dilution of the urine. Results of Multiplex ELISA were normalized to creatininuria (concentration of the complement protein/ concentration of urinary creatinine, giving a ratio of ng or µg of complement protein/mmol of creatinuria). Normalization to creatininuria mitigates the effects of urine concentration. In addition, we calculated the activation fragments to native proteins ratio. This aimed to distinguish activation from presence of complement proteins, the latter being influenced by proteinuria levels. Ba/C3 ratio was calculated because there is no intact FB detection in the kit. Specificity for C5a/C5 ratio was calculated as the ratio of the number of true negatives to the sum of numbers of true negatives and false positives.

### Glycerol-induced rhabdomyolysis model and mouse treatment

RIAKI was induced by intramuscular injection of glycerol in C57Bl/6 male mice^8^. Wild type, male C57BL/6 mice at 6- to 10-weeks of age were purchased from Charles River Laboratories. Experimental protocols were approved by the Charles Darwin ethical committee (Paris, France) and by the French Ministry of Agriculture (Paris, France number APAFIS#2148 2019091015099240v1). All the experiments were conducted in accordance with their recommendations for care and use of laboratory animals. We have complied with all relevant ethical regulations for animal use. In order to induce rhabdomyolysis, mice were injected intramuscularly in the left quadriceps by 200 µL of glycerol 50% as previously established^8^. All mice were sacrificed by cervical dislocation 1 day after glycerol administration except for the kinetic model where they were sequentially at hour (H) H1, H3, H12, H24 and day (D) D7. The dose and timing for the use of complement inhibitors were selected based on the literature. 1000 µg/mouse of BB5.1 was injected 2 h before glycerol injection and compared to PBS^52^. PMX53 (purchased from Tebubio in one experiment and kindly provided by Prof. Trent Woodruff, University of Queensland, Herston, QLD, Australia, for another one) was administrated intraperitoneally at 1 mg/kg at H-12, H0 and H + 12 after rhabdomyolysis induction^53^. The efficacy of muscle injury was assessed in mice by the elevation of ASAT, more reliable parameter in mice compared to CK. Whole blood was collected retro-orbitally with heparinized capillary tube in microtubes with 10 µL EDTA. Microtubes were centrifuged at 12,000 × *g* for 10 min at room temperature to recover plasma. Plasma was directly frozen at −80 °C. Kidneys were immediately snap frozen in liquid nitrogen for immunofluorescence (IF), molecular biology and freshly recovered by flow cytometry analysis. For glycerol injection and sacrifice, mice were anesthetized with isoflurane 2–3%. If mice in any experiment had to be excluded from analysis, it was because of technical issues with sample collection or handling. For PBS or Glycerol injection mice were randomized by randomly injecting the mice with each treatment and placing mice injected with both treatments in the same cage to avoid cage effect. One person prepared each mouse and the injection syringe and another person, blinded for the content of the syringe, injected the mice. The experiment was performed in the morning and the animals were observed at midday and in the late afternoon for the 24 h experiments and daily for the 7 day experiment. Mice were kept in groups of 2–5 and housed in appropriate Type IIL cages offering sufficient living space, on racks with individual thermoregulated ventilation (22 °C, +/−2 °C), 12-h lighting (daytime phase from 7 a.m. to 7 p.m.) and automatic watering. An enriched environment suitable for housing mice is provided. Human endpoint included moribund/unresponsive, non-ambulatory, or unable to reach food/water and recumbency. Only male mice were used, as female mice have weaker complement activation capacity and develop less severe complement-mediated kidney diseases, as well as RIAKI^54–56^. The choice of the experimental mouse strain and the methodology is standard and recapitulates relatively well the human pathology.

### Evaluation of the biochemical parameters of mice and patients

For mice, kidney function and muscle injury were evaluated by plasmatic Blood Urea Nitrogen, Creatinine, Creatine Kinase (CK) and Aspartate AminoTransferase (ASAT) coupled with the liver injury marker Alanine AminoTransferase (ALAT) using Konelab equipment. Likewise, creatinine levels were measured in patients’ urine by Konelab Chemistry Analyzer equipment (Thermo Scientific).

### Gene expression analysis by QuantiGene

Thirty-μm-thick frozen sections of snap frozen whole mouse kidneys were cut with a Cryostat at −20 °C (Leica AS-LMD, Leica Biosystem) and homogenized in 200 μL of 1-Thioglycerol/Homogenization Solution (QuantiGene®, ThermoFisher Scientific). A panel of 80 genes - 5 housekeeping genes and 75 chosen from RIAKI transcriptomic signature, previously determined by RNAseq^8^ (Supplementary Table 3). Target genes were submitted to targeted hybridization and signal amplification according to the recommendation of the manufacturer. Streptavidin phycoerythrin signal was detected by Luminex equipment (Luminex Corporation). Blank well fluorescence was subtracted from median fluorescence and housekeeping genes validated regarding their standard deviation to mean ratio. Analysis was performed with Graphpad Prism® software after normalization of mean fluorescence values on housekeeping gene expression and comparison with gene expression of pooled PBS treated mice, according to the manufacturer’s instructions.

### Histology

The histology was assessed by hematoxylin/eosin staining on 5-µm-tick sections of paraffin-embedded kidneys. After dewaxing in Clearene (Leica, Clearene 5 L 3803600E) and rehydration with gradual concentration of ethanol (100%, 90%,70% and 50%), hematoxylin and eosin staining was performed by routine procedures. The slides were scanned with a Nanozoomer whole slide scanner (Hamamatsu Photonics) and blindly quantified. Tubular dilatation and casts were given a score between 0 and 3, which described the percentage of the total cortical area of the biopsy (0 = 0–2%, 1 = > 2–25%, 3 = > 25–50%, and 4 > 50%).

### Immunofluorescence (IF) and immunochemistry (IHC) in mice

Frozen kidneys were cut at 5-µm-thick sections with a Cryostat at −20 °C (Leica AS-LMD, Leica Biosystem) and fixed in acetone on ice for 10 min. 4-µm-thick sections of paraformaldehyde-fixed and paraffin-embedded kidneys were cut with a Microtome (Thermo Scientific Microm HM 340E) for IHC. After dewaxing in Clearene (Leica, Clearene 5 L 3803600E) and rehydration with gradual concentration of ethanol (100%, 90%,70% and 50%), endogenous peroxidases were blocked with 3% H_2_O_2_ (Gilfrer, 10603051) and non-specific staining by protein block (Dako, X0909). A rat anti mouse C3b/iC3b antibody (HM1065, Dako, 1 µg/ml) was incubated for 30 minutes and revealed by a chicken anti rat IgG H + L AF647 (A21247, Invitrogen) or a Chicken anti-rat IgG H + L AF488 (A21470, Invitrogen). A double immunostaining was performed with an anti CD31 (NCL- CD31- 1A10, Leica, 36 µg/L), revealed by an anti-mouse Polyview Plus AP and AF 647 Tyramid reagent Invitrogen, B40958. Mounting was performed with ProLong antifade reagent (Invitrogen). TUNEL assay was performed with the DeadEND TM Fluometric TUNEL (Terminal deoxynucleotidyl transferase dUTP Nick-End Labeling) system G3250 kit from Promega, according to manufacturer’s recommendations. Slides for IF were scanned with Axio Scan™ Z1 (Zeiss, Oberkochen, Germany). Images were analyzed and staining were quantified using Halo software (Indica Labs).

### Multiplexed sequential immunofluorescence (seqIF) and microscopy

Hyperplex immunofluorescence imaging was achieved by the automated multiplexed seqIF staining^57^ on frozen kidney sections from 2 mice injected with glycerol and 2 mice injected with PBS were examined using the COMET platform (Lunaphore Technologies). Hyperplex immunofluorescence imaging, applying our in situ Complementomics approach^16^, was also performed on FFPE sections from five patients with RIAKI, in comparison to a peri-tumoral tissue from clear cell renal cell carcinoma. The multiplexed panel consisted of 6 antibodies, including both cell-specific markers and complement antigens for human biopsies (extracted from our in situ Complementomics panel^16^) and 5 antibodies for mice (Supplementary Tables 4, 5). All antibodies were validated using conventional immunohistochemistry (IHC) and immunofluorescence (IF) staining in conjunction with corresponding fluorophores and DAPI (ThermoFisher Scientific). Secondary antibodies Alexa Fluor 555 (A32727, ThermoFisher Scientific) and Alexa Fluor 647 (A32733, ThermoFisher Scientific) were used at dilutions of 1:200 and 1:400, respectively. Acquired images were analyzed using Halo Image Analysis software (Indica Labs) proximity analysis algorithm for inter cells distance quantification, setting a threshold at 10 micrometer to quantify the number of CD11b+ cells within 10μm from C3b/iC3b positive tubular cells (double positive for C3b/iC3b and Na/K-ATPase). Only 5 biopsies were tested as these tissues are extremely rare, RIAKI patients are not usually biopsied. No urine samples were available for these patients.

### Evaluation of the myeloid infiltrate in mouse kidneys by flow cytometry

24 h after injection of PBS or glycerol in vivo, mice were sacrificed and the kidneys were decapsulated and kept in RPMI1640 Medium (Gibco, Life technologies 12633012) on ice. Kidneys were then cut into small piece and incubated in a mix of enzymes from Multi Tissue Dissociation Kit 3 (Miltenyi Biotec) within an Octodissociator (Miltenyi Biotec) using the appropriate program (37C_Multi_E) then filtered on a 70 µm nylon mesh (Miltenyi Biotec). Cells in suspension were counted using LUNA® technology before incubation with FcBlock (anti-CD16/34, clone 2.4G2, 10 µg/ml). Cells were incubated for 30 min with conjugated antibodies from Supplementary Table 6, then washed by PBS/FCS and suspended in 4% paraformaldehyde (PFA). Stained cells with capture of 2.5 million events per samples were analyzed by BD Fortessa flow cytometer (BD Bioscience) and analyzed by FlowJo software (BD Bioscience) to identify neutrophiles (LD−/CD45+/CD11b+/CD11c−/SSC high/LY6G+), inflammatory monocytes (LD−/CD45+/CD11b+/CD11c−/SSC low/LY6G−/Ly6C high), eosinophils (LD−/CD45+/CD11b+/CD11c−/SSC high/LY6G−) and resident macrophages (LD−/CD45+/CD11b+/CD11c−/LY6G−/Ly6C low). The gating strategy is presented at Supplementary Fig. S10.

### Estimation of myeloid infiltrate in mouse kidney by mMCP-counter

To evaluate the proportion of immune cells infiltrating the kidney, we also used a recently developed method, the mouse Microenvironment Cell Population counter (mMCP-counter), which is based on highly specific transcriptomic markers that accurately quantify immune (and stromal) murine cell populations^58^. We used the QuantiGene method (QuantiGene panel, Supplementary Table 3) to quantify these transcriptomic markers of frozen kidney, and ran the mMCP-counter with the normalized matrix of mean fluorescence to evaluate the relative abundancy of immune cell populations in the tissue (https://github.com/cit-bioinfo/mMCP-counter)^58^.

### Complement C3 evaluation in mouse urine using Western Blot

Urine samples were used pure. All samples were prepared using NuPAGE® LDS sample buffer (4X) from Thermo Fisher Scientific, along with a reducing agent (DTT 1M), and subsequently denatured at 90 °C for 10 min. Protein separation was carried out on a NuPAGE 10% Bis-Tris gel (Thermo Fisher Scientific). The proteins were then transferred onto a nitrocellulose membrane using the iBlot system from Invitrogen. Following this, the membranes were subjected to overnight incubation with primary antibody (Goat IgG fraction anti-mouse complement C3, MP BIOMEDICALS, #55463). Subsequently, they were incubated with a secondary antibody (rabbit anti-goat HRP, Thermo Fisher Scientific, #31402 for C3 and goat anti-rabbit HRP, Thermo Fisher Scientific, #31460). Visualization was achieved through chemiluminescence using a substrate for HRP (SuperSignal™ West Dura Luminol, Thermo Fisher Scientific, #1856145), detected using the iBright Western Blot Imaging System (iBright FL1500, Thermo Fisher Scientific). The raw western blot image is given as Supplementary Fig. S11.

### Complement C3 and C3a evaluation in mouse urine using ELISA

C3 in the urine was quantified using C3 mouse ELISA (Hycult, Catalog #HK2002) and C3a – using Mouse C3a ELISA Kit (Invitrogen, ThermoFisher Scientific, Catalog #EEL091). The quantification was done following manufacturer instructions. Urine was diluted 1/5 for the C3 and 1/2 for C3a for the urine quantification.

### Statistics and reproducibility

Results were analyzed using the statistical software GraphPad Prism 9 (La Jolla, USA) and with R for Kruskall–Wallis. Two continuous variables were compared using the Mann–Whitney test. Comparisons between more than 2 groups of mice, were performed using Kruskal–Wallis test, followed by Dunn’s test with an accepted alpha risk of 0.05 and a Benjamini–Hochberg adjustment method for multiple pairwise comparisons. Statistical significance was defined as *p* < 0.05, with bilateral tests.

The mouse experiment comparing PBS vs GLY injected mice was performed 3 times and the results are pooled, whenever the measurement was performed in more than one experiment. Total of 14 PBS and 22 GLY injected mice were used for this comparison. Overall, two times more GLY-injected mice than PBS were used, because of the variability of the model. Kinetic study was performed once and mice were sacrificed at 0, 1, 3, 12, 24 hours (*n* = 5/time point) and 7 days (*n* = 3) after injection by GLY.

## Supplementary information

Supplementary Information

Description of additional supplementary file

Supplementary Data 1

Featured image

## Data Availability

The source data and datasets supporting the conclusions of this article are included within the article and its supplementary files, including in the Supplementary Data 1. The clinical data of the patients is available in Supplementary Tables 1 and 2. All other data are available from the corresponding author.
