# Supplementary material for: The temporal gene expression landscape of rhabdomyolysis-induced acute kidney injury reveals the timing of complement activation
Source: Commun Biol. 2025 Dec 31;9:171. doi: 10.1038/s42003-025-09449-y (PMC12877018; doi:10.1038/s42003-025-09449-y)
Supplement: Supplementary file 1 — Supplementary Information [file 42003_2025_9449_MOESM1_ESM.pdf]

Supplementary Figures and Tables

The temporal gene expression landscape of rhabdomyolysis-induced acute kidney injury reveals the timing of complement activation

Anne Grunenwald, Idris Boudhabhay, Margot Revel, Victoria Poillerat, Elodie Voilin, Amine Majdi, Khalil Chaibi, Stephane Gaudry, Trent M Woodruff, Gilles Crambert, Julien Guihaire, Mohamad Zaidan, Julie Oniszczuk, Marie Frimat, Viviane Gnemmi, Marc Aletti, Hubert Nielly, Laurent Gilardin, Lubka T. Roumenina

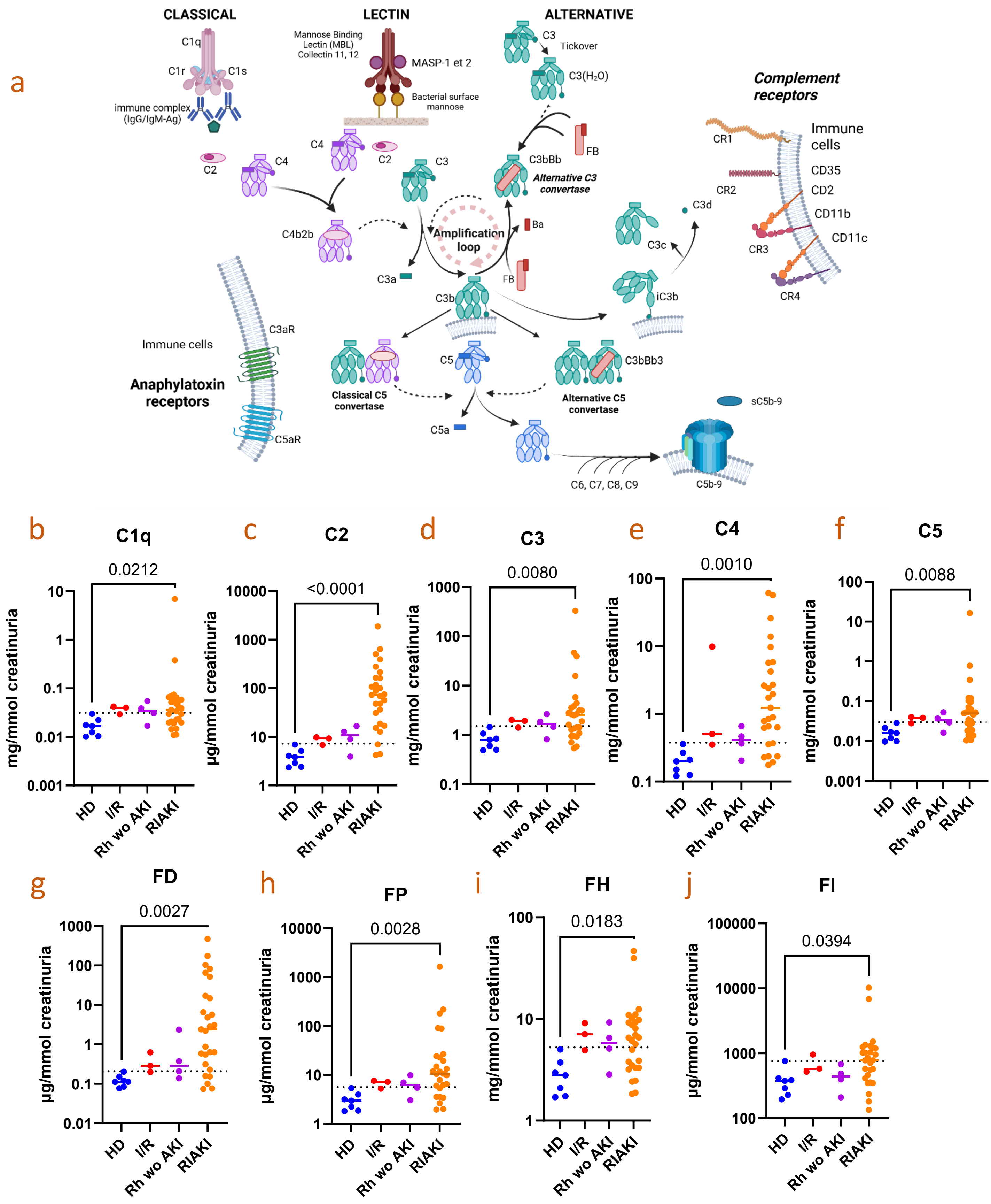

Supplementary Figure 1: Intact complement proteins in the urine of RIAKI patients. (a) Schematic representation of the complement system to illustrate the position in the cascade of all complement components explored in this study. Figure created with Biorender.com, https://BioRender.com/chpkukp. **(b-j)** Complete complement proteins in urines of healthy donors (HD, n=7), patients with renal ischemia reperfusion (IR, n=3), rhabdomyolysis (Rh) without (wo) acute kidney injury (AKI), n=4, or Rhabdomyolysis induced AKI (RIAKI, n=27): C1q **(b)**, C2 **(c)**, C3 **(d)**, C4 **(e)**, C5 **(f)**, Factor D (FD) **(g)**, Properdin (FP) **(h)**, Factor H (FH) **(i)**, Factor I (FI) **(j)**, Kruskal Wallis test with Dunn's correction for multiple comparisons to HD (healthy donors). The normal level cut-off, calculated as the average +2SD of the healthy donors is indicated as a dotted line).

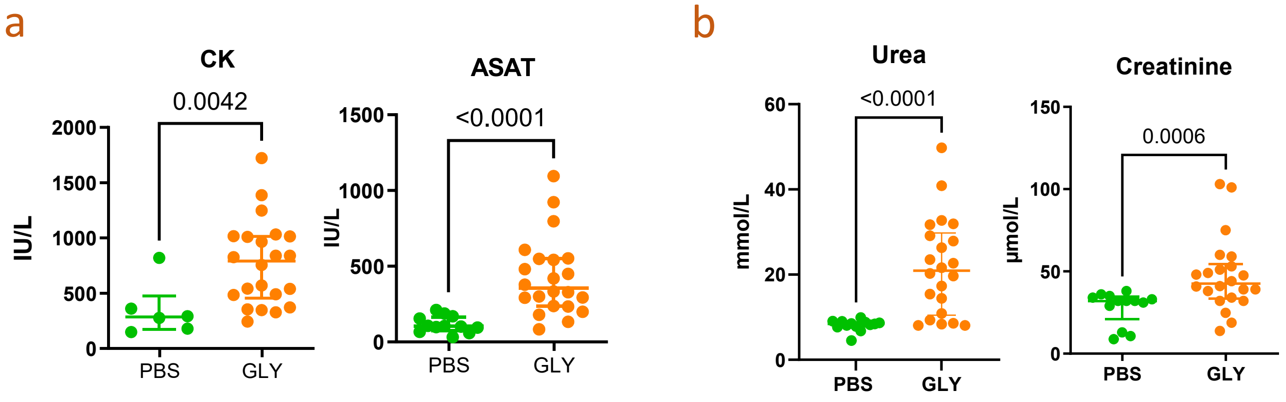

Supplementary Figure 2: Characterization of renal injury, muscular phenotype 24 hours after mice injection by PBS or glycerol (GLY) (a) Muscle injury estimated by Creatin Kinase (CK, left) and Aspartate-Amino-Transferase (ASAT, right) levels (IU/l) at 24 hours in mice injected by PBS or GLY. **(b)** Renal function estimated by urea (mmol/l) and creatinine (µmol/L) concentrations at 24 hours in mice injected by PBS or GLY. Mann-Whitney test.

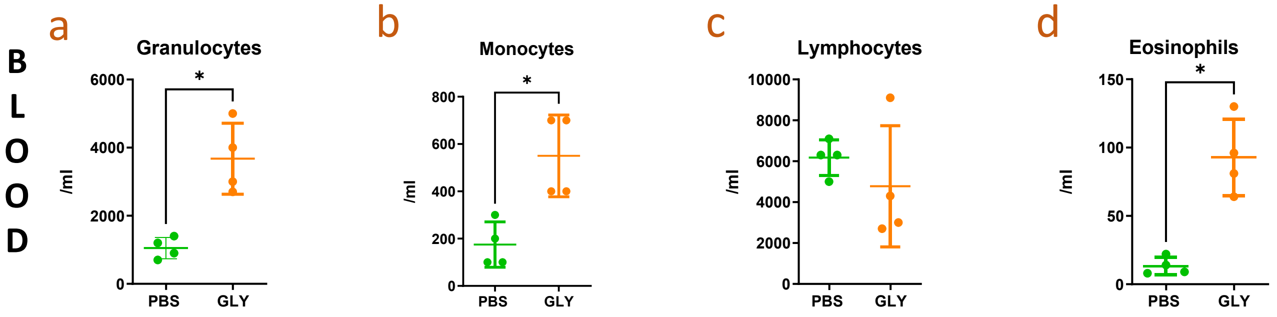

Supplementary Figure 3. Blood immune cell counts in the RIAKI mice. Variation in granulocytes **(a),** monocytes **(b),** lymphocytes **(c),** eosinophils **(d)** counts by Blood counter in blood-EDTA samples in C57Bl6 WT mice injected by PBS or Glycerol. The presented results are from one experiment with 4 PBS and 4 GLY injected mice.

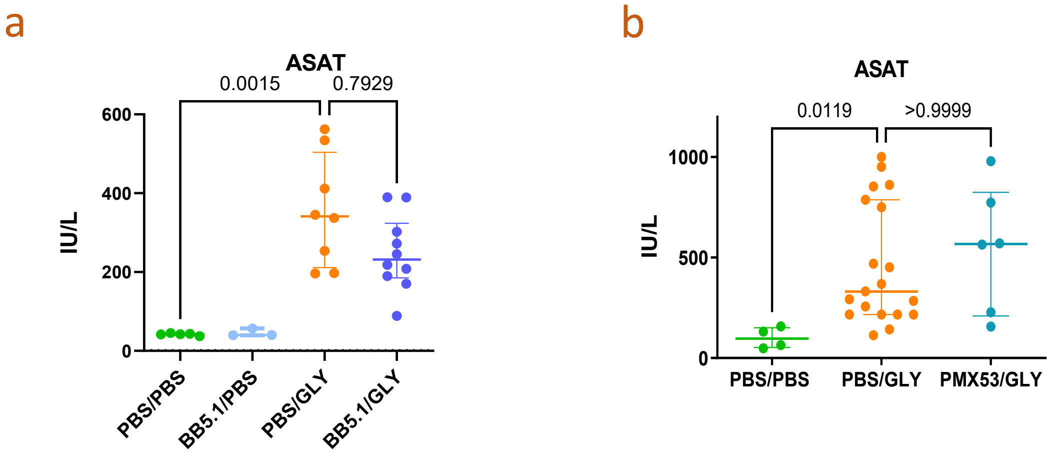

Supplementary Figure 4: Muscle injury in mice injected by PBS or glycerol (GLY) with or without complement inhibitors (a) C5aR1 inhibitor PMX53 (1mg/kg, diluted in PBS, intraperitoneal injection 12 hours before, concomitantly and 12h after glycerol injection); **(b)** C5 inhibitor BB5.1 (1000µg/mouse diluted in PBS, intravenous, 2 hours before glycerol injection). Kruskal Wallis test with Dunn's correction for multiple pairwise comparisons.

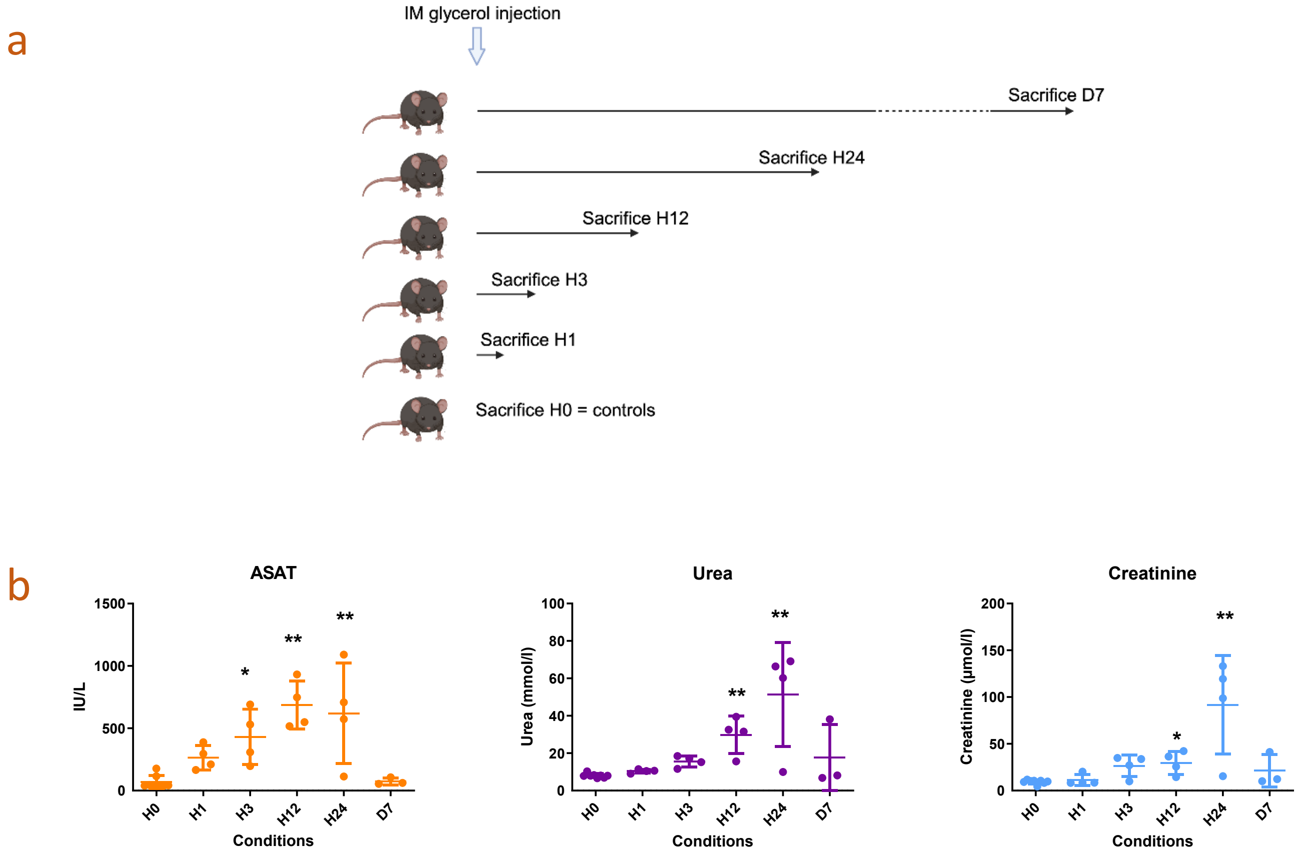

Supplementary Figure 5: Characterization of renal injury, muscular phenotype (a) Schematic representation of the kinetic RIAKI experiment. Mice were injected intramuscularly with glycerol and sequentially sacrificed at 1 hour (H1), H3, H12, H24 and day 7 (D7). **(b)** Plasma ASAT (orange), urea (violet), creatinine (light blue) levels from mice injected by glycerol at H0, H1, H3, H12, H24 and D7. Different time points were compared to control condition (H0). * $p<0.05$, ** $p<0.01$; Kruskal Wallis test with Dunn's correction for multiple pairwise comparisons. Presented results are from one experiment with 5 mice at time 0, 4 mice at 1 hour, 3 hours, 12 hours and 24 hours each, and 3 mice at day 7.

a

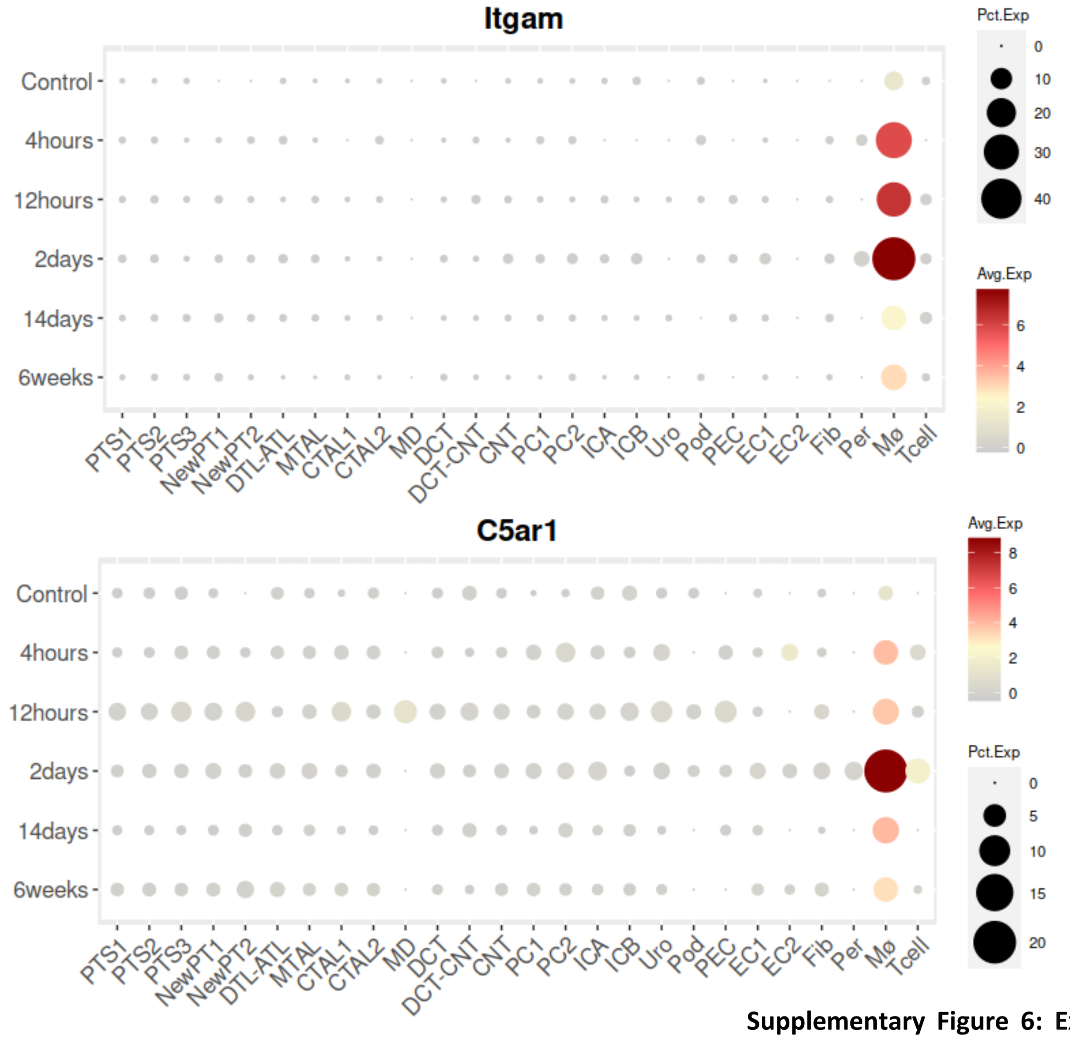

b

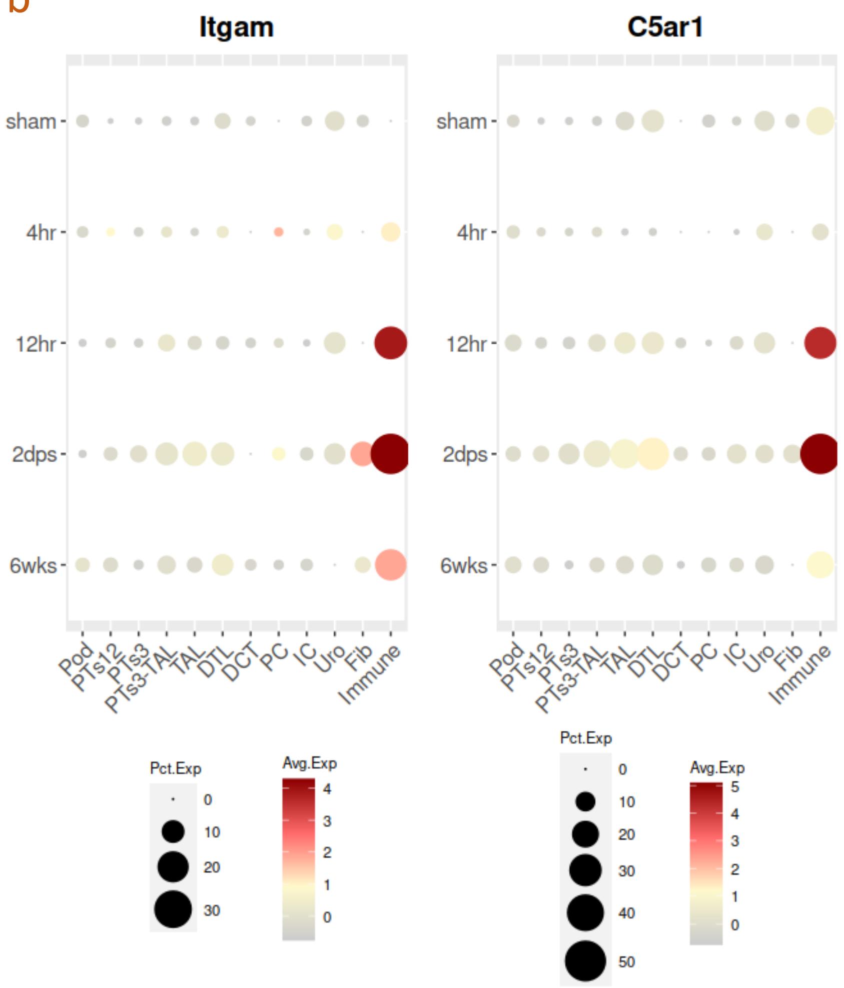

Supplementary Figure 6: Expression profiles of complement receptors in murine kidney RNA sequencing datasets following ischemia-reperfusion injury. (a) Analysis of *Itgam* and *C5ar1* expression from the Kirita et al. dataset[24], which involved single nuclei RNAseq data. **(b)** Comparative analysis of *Itgam* and *C5ar1* expression from the Dixon et al. dataset[23], using single cell RNAseq data. The data were generated using the Kidney Interactive Transcriptomics Analyzer. https://humphreyslab.com/SingleCell/

ATL, thin ascending limb of loop of Henle; Bil, bilateral; CNT, connecting tubule; CPC, principle cells ofcollecting duct in cortex; CTAL, thick ascending limb of loop of Henle in cortex; DCT, distal convoluted tubule; DTL, descending limb of loop of Henle; EC,endothelial cells; Fib, fibroblasts; ICA, type A intercalated cells of collecting duct; ICB, type B intercalated cells of collecting duct; MD, macula densa; Mø,macrophages; MPC, principle cells of collecting duct in medulla; MTAL, thick ascending limb of loop of Henle in medulla; PEC, parietal epithelial cells; Per,pericytes; Pod, podocytes; PT-S1, S1 segment of proximal tubule; PT-S2, S2 segment of proximal tubule; PT-S3, S3 segment of proximal tubule; Uro, urothelium.

Pct.Exp : Percentage of Expression, Avg.Exp : Average of expression

a

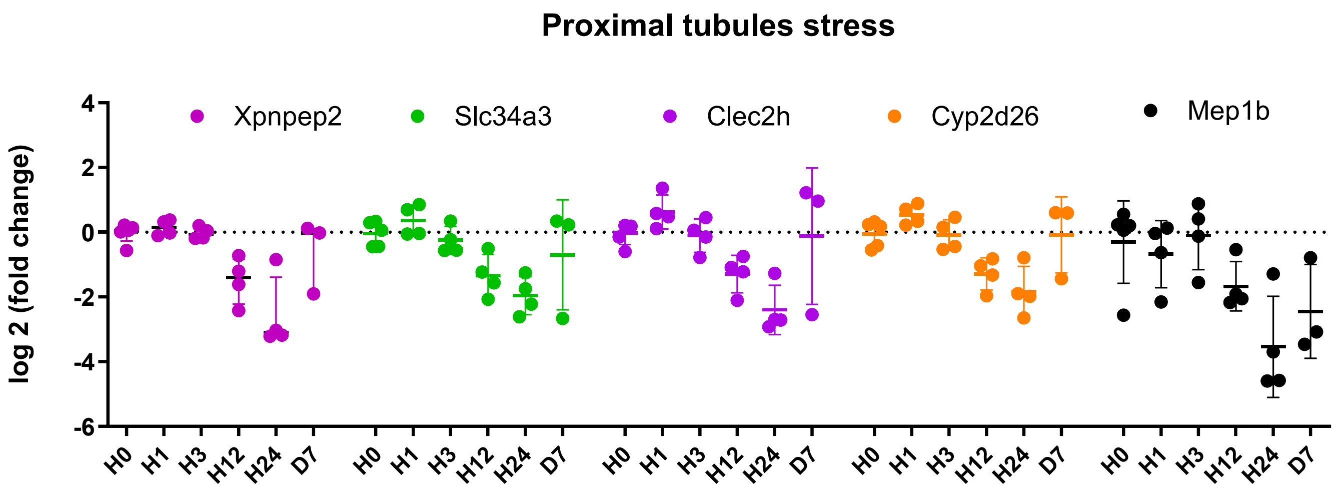

b

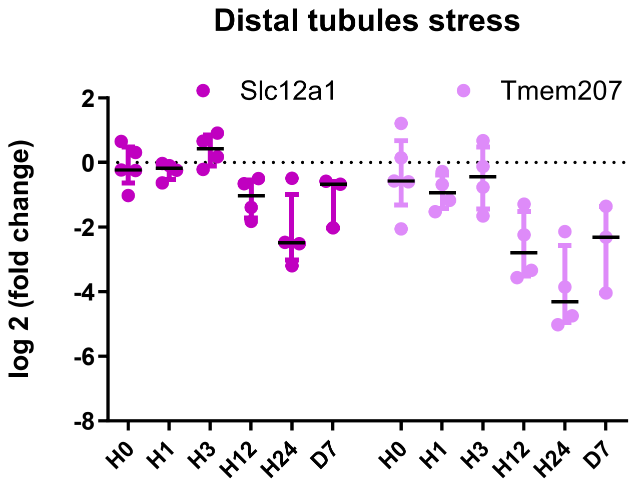

Supplementary Figure 7: Kinetic of loss of tubular cell markers during RIAKI. Tubular stress assessed by the loss of several markers determined from the RIAKI signature **affecting both** proximal **(a)** - *Xnpep2* (X-Prolyl Aminopeptidase 2), *Slc34e3* (Sodium-dependent phosphate transport protein 2C), *Clec2h* (C-type lectin domain family 2 member H), *Cyp2d26* (Cytochrome P450 2d26) and *Mep1b* (Meprin A Subunit Beta) - , and distal **(b) -** *Slc12a1* (Na+/K+/2Cl- cotransporter, NKCC2) and *Tmem207* (Transmembrane Protein 207) evaluated by QuantiGene on snap-frozen kidney at H0, H1, H3, H12, H24 and day 7 after injection by glycerol - tubules.

.

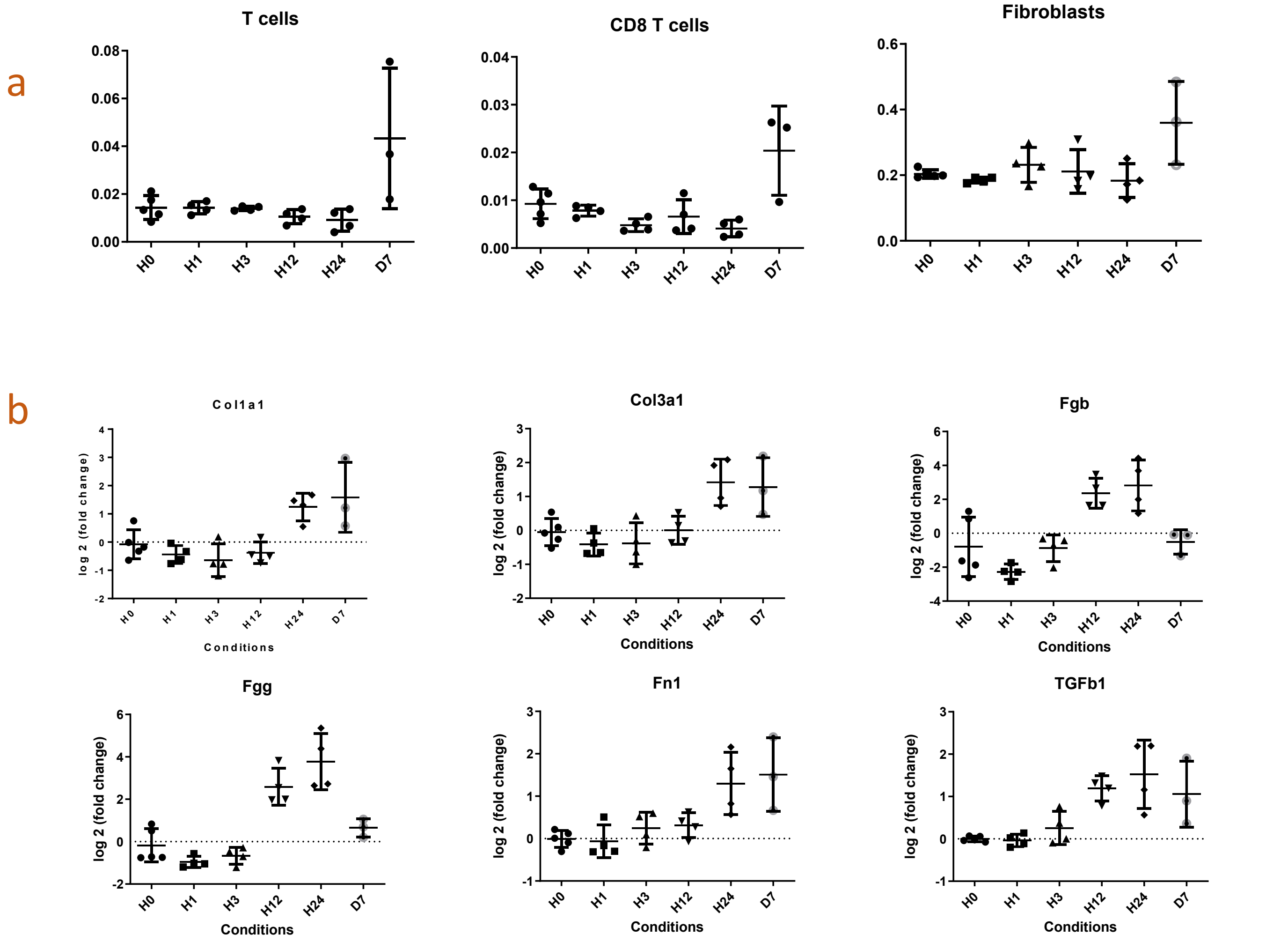

Supplementary figure 8: Fibrosis/repair pattern in RIAKI mice. (a) Estimation of relative abundancy of T cells (CD3+), CD8 T cells (CD8+) and Fibroblasts from normalized QuantiGene median values of genes analyzed with mMCP algorithm **(b)** Differential expression of *Col1a1* (Collagen, type I, alpha 1 - up left), *Col3a1* (Collagen, type III, alpha 1 - up middle), *Fgb* (Fibrinogen beta chain – up right) and *Tgfb1* (Transforming growth factor beta 1 - bottom right), *Fn1* (Fibronectin 1 – bottom middle), *Fgg* (Fibrinogen gamma chain – bottom right) by QuantiGene in snap-frozen kidney of C57Bl6 WT mice at H0, H1, H3, H12, H24 and D7 after injection by glycerol. Presented results are from one experiment with 5 mice at time 0, 4 mice at 1 hour, 3 hours, 12 hours and 24 hours each, and 3 mice at day 7.

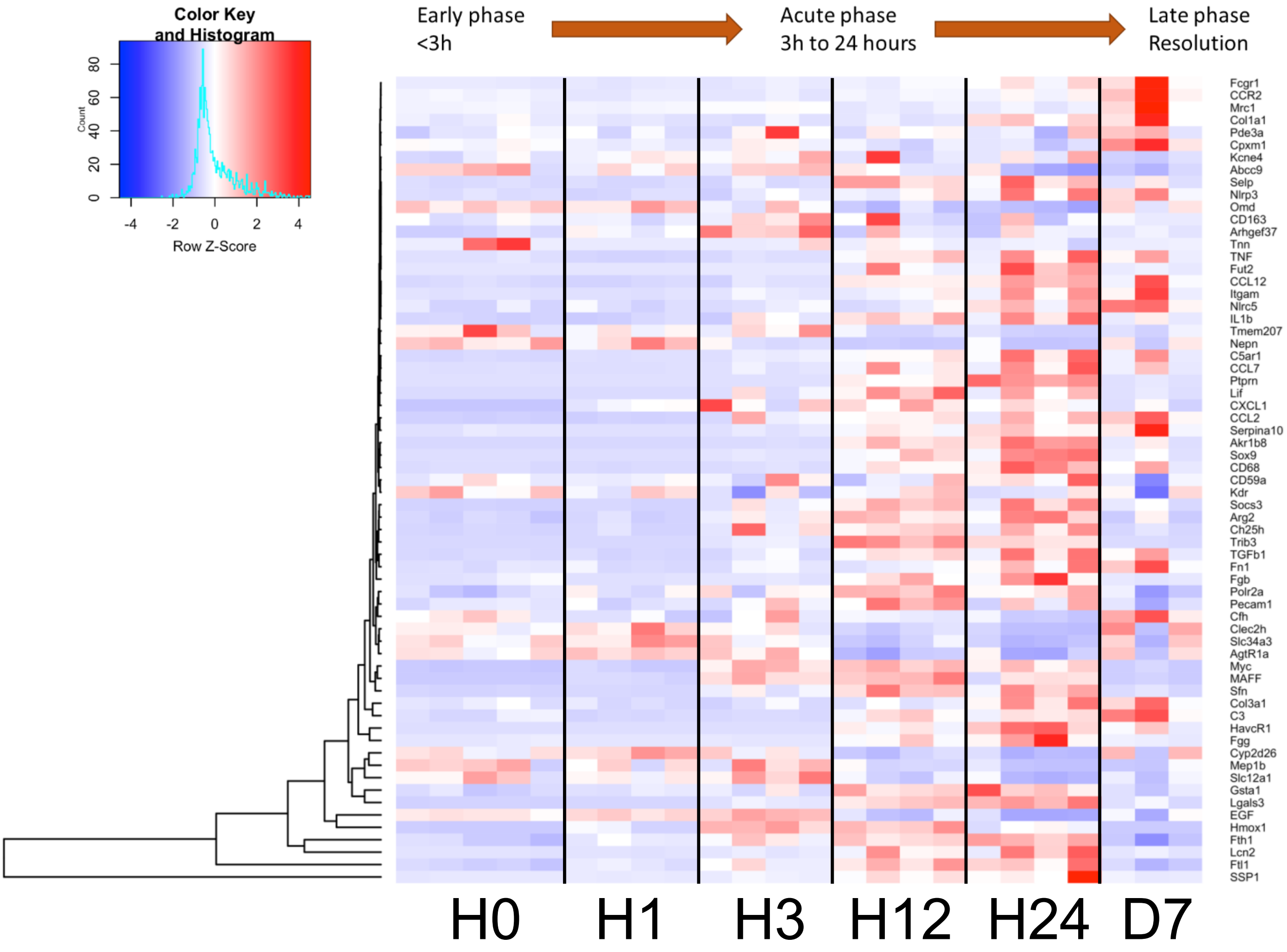

Supplementary figure 9: Global representation of the transcriptomic changes at different time points of RIAKI, evidenced with our selected gene set**.**

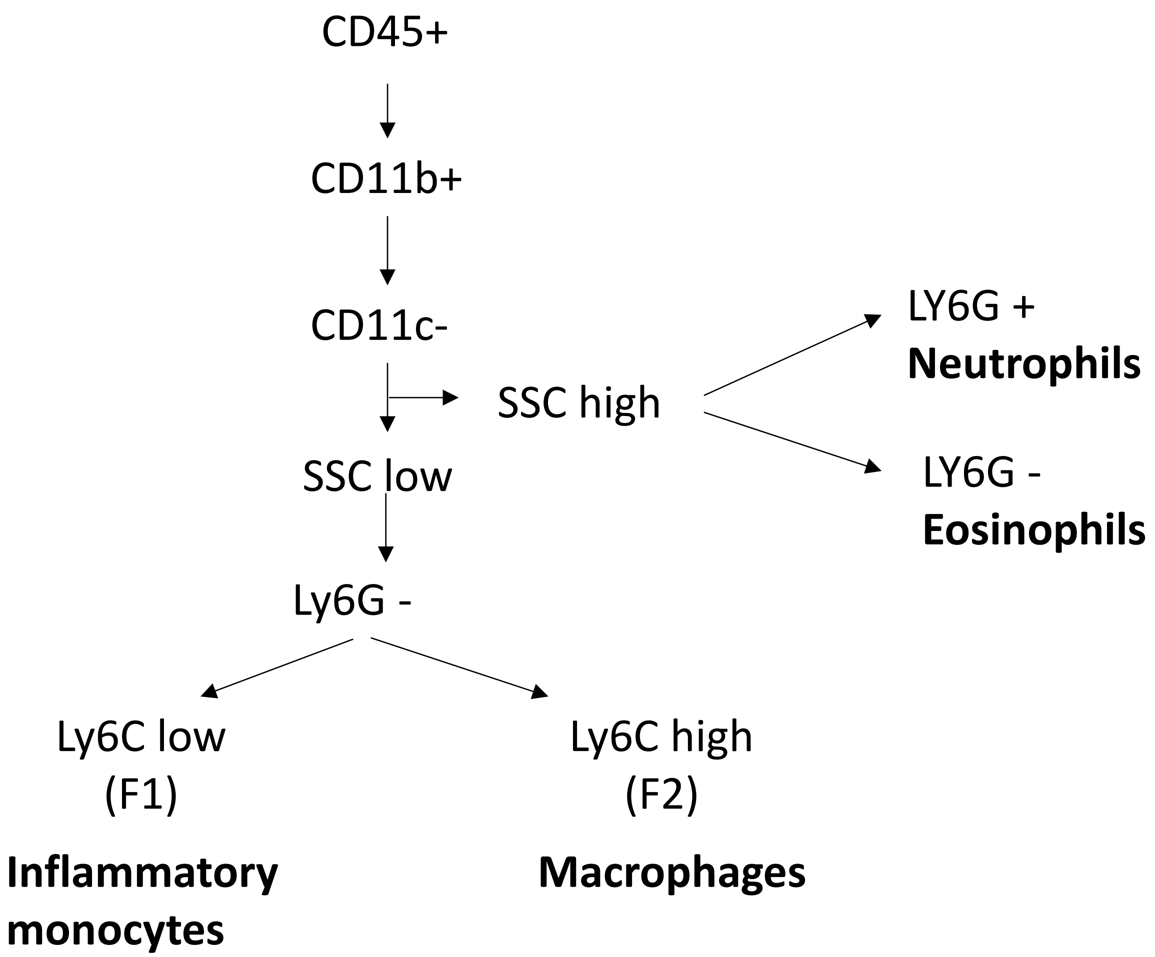

Supplementary figure 10: Gating strategy for the flow cytometry. Gating workflow for the analysis of kidney immune cell populations in glycerol-injured and control mice

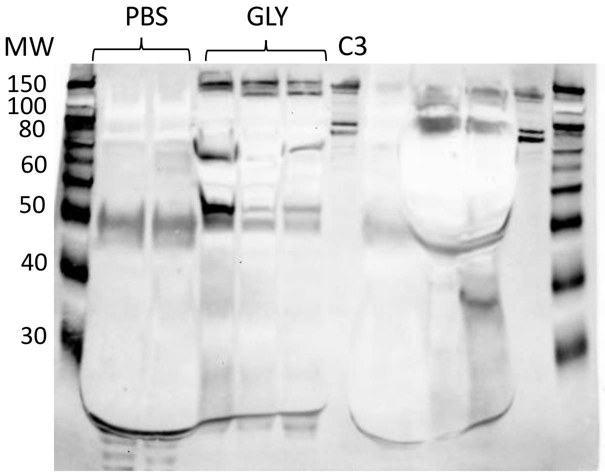

WB from Figure 3c

Supplementary figure 11: Raw image of the western blot from Figure 3c

Supplementary Tables

Supplementary Table 1: Clinical data when available for the RIAKI urine cohort. AHT: arterial hypertension, AKI: acute kidney injury, CPK: creatine phosphokinase (in IU/L), Creatinine in µmol/l

P	Sex	Age	AHT	Diabetes	Smoke	AKI	CPK at admission	Circumstances	Initial creatinine
1	M	>75	Yes	No	No	Yes	6000	Muscle compression	94
2	F	50-75	No	No	No	Yes	7800	Muscle compression	61
3	M	25-50	No	No	No	No	59000	Strenuous exercise	92
4	M	>75	Yes	Yes	No	Yes	18000	Muscle compression	140
5	M	50-75	No	Yes	No	No	31662	Muscle compression	46
6	M	>75	Yes	No	No	Yes	3900	Muscle compression	179
7	M	<25	No	No	No	No	96000	Strenuous exercise	na
8	F	>75	No	No	No	Yes	5014	Muscle compression	na
9	M	50-75	Yes	Yes	No	Yes	1095	Sepsis	113
10	M	<25	No	No	No	Yes	1564	Strenuous exercise	166
11	M	50-75	No	No	Yes	Yes	4022	n.a.	na
12	M	>75	No	No	No	No	>20000	Muscle compression	87
13	F	50-75	Yes	Yes	No	Yes	12569	Toxic	na
14	M	25-50	No	No	No	Yes	10 000	Mac Ardle disease	1615
15	M	<25	No	No	No	Yes	na	na	na
16	M	25-50	No	No	No	Yes	5000	Strenuous exercise	132
17	M	50-75	Yes	No	Yes	Yes	>1000	Sepsis	500
18	M	>75	Yes	No	No	Yes	>1000	na	na
19	F	50-75	Yes	No	Yes	Yes	>1000	na	513
20	M	50-75	No	No	No	Yes	>1000	Sepsis	259
21	M	25-50	No	Yes	No	Yes	>1000	na	364
22	M	50-75	Yes	No	No	Yes	>1000	na	370
23	M	50-75	Yes	No	Yes	Yes	>1000	na	334
24	F	>75	Yes	No	No	Yes	>1000	na	301
25	F	50-75	No	No	No	Yes	>1000	na	215
26	F	50-75	Yes	No	No	Yes	>1000	na	249
27	M	50-75	Yes	Yes	No	Yes	>1000	na	531
28	M	50-75	Yes	No	Yes	Yes	>1000	na	379
29	F	50-75	Yes	No	No	Yes	>1000	na	269
30	M	50-75	Yes	No	No	Yes	>1000	na	361
31	M	50-75	No	No	No	Yes	>1000	na	254
32	H	50-75	Yes	No	Yes	Yes	>1000	na	703

Supplementary Table 2: Clinical data when available for the RIAKI patients with kidney biopsies used for Multiplexed sequential Immunofluorescence. AHT: arterial hypertension, AKI: acute kidney injury, CPK: creatine phosphokinase (in UI/L), Creatinine in µmol/l, NA: not available.

P	Sex	Age	AHT	Diabetes	Smoke	CPK at admission	Circumstances	Initial creatinine	AKI
A	M	<25	No	No	No	10100	Muscle compression	130	Yes
B	M	25-50	Yes	No	Yes	15000	Muscle compression	450	Yes
C	M	50-75	No	No	No	1500	Muscle compression	210	Yes
D	M	50-75	Yes	No	No	1698	NA	780	Yes
E	M	50-75	No	No	No	461000	Dermatomyositis	412	Yes

Supplementary Table 3: 80 plex panel for quantigen designed after RIAKI signature [1]

**Genes**					
Ccl12	Mep1b	Omd	Cd3e	Fut2	Xpnpep2
Ccl2	Nlrp3	Tmem207	Cd6	Havcr1	Lif
Ccl7	Nlrc5	Kdr	Cd8b1	Myc	Clec2h
Col1a1	Slc12a1	Pecam1	Nepn	Lcn2	Arg1
Col3a1	Slc34a3	Selp	Ptprn	Gsta1	Arg2
C3	Sox9	Sfn	Trib3	Arhgef37	Kcne4
C5aR1	Ch25h	Fgb	Maff	Fcgr1	Rarres2
CD59	Cyp2d26	Fgg	Actb	Ccr2	Hmox1
Cfh	Akr1b8	Fn1	Gapdh	Lgals3	Ceacam10
Crry	Cd163	Serpina10	Rsp3	Spp1	Mrgpra2b
Cxcl1	Cd68	Tnn	Rps18	Pde3a	
Il1b	Itgam	Abcc9	Pol2ra	Egf	
Il6	Mrc1	Agtr1a	Fth1	Tgfb1	
Tnf	Socs3	Cpxm1	Gusb	Ftl1	

<u>Supplemental Table 4:</u> Antibodies and experimental conditions for Multiplexed Sequential Immunofluorescence in human FFPE kidney biopsy.

Antibody	Species	Clone	Supplier	Dilution
**Megalin** *(proximal tubule cells)*	Rabbit		Abcam	1:2000
**CD31** *(endothelial cells)*	Goat	Polyclonal	Bio-Techne	1:50
**C3b/iC3,**	Rat	2/11	Hycult	1:100
**cCaspase3** *(cell death)*	Rabbit	5A1E	Cell Signaling Technology	1:100
**CD11b** *(CR3/myeloid cells)*	Rabbit	EPR1244	Abcam	1:500

<u>Supplemental Table 5:</u> Antibodies and experimental conditions for Multiplexed Sequential Immunofluorescence in frozen mouse kidney.

Antibody	Species	Clone	Supplier	Dilution
**Na+K+ ATPase** *(tubule cells)*	Rabbit	EP1845Y	Abcam	1:2000
**CD34** *(endothelial cells)*	Mouse	QBend-10	Dako	1:100
**C3c**	Rabbit	Polyclonal	Dako	1:500
**C3d**	Rabbit	Polyclonal	Dako	1:1000
**FH**	Rabbit	Polyclonal	ProteinTech	1:100
**CD11b** *(CR3/myeloid cells)*	Rabbit	EPR1244	Abcam	1:500

<u>Supplementary Table 6:</u> Cytometry antibodies and life marker panel. BP: band pass. YG: yellow-green.

Laser	BP Filter (nm)	Channel	Antigen	Fluoro-chrome	Dilution	Clone	Reference	Type
Blue	530/30	Blue B	**C3aR**	FITC	1:50	74	MA5 17475, Invitrogen	Mouse IgG1
	710/50	Blue A	**CD206**	PerCP Cy5.5	1:50	C068C2	141701, Biolegend	Rat IgG2aK
Violet	450/50	Violet F	**IA IE**	eF450	1:1000	M5/114	MA1-10403, Invitrogen	Rat IgG2b
	525/50	Violet E	**LD**	LD	1:200	Aqua	L34957, Termofischer	
	610/20	Violet D	**Ly6C**	BV605	1:100	HK1.4	128036, Biolegend	Rat IgG2c, κ
	780/60	Violet A	**CD11b**	BV785	1:400	M1/70	101243, Biolegend	Rat IgG2b, κ
Red	670/30	Red C	**C5aR1**	APC	1:200	20/70	135808,	Rat IgG2b, κ
	730/45	Red B	**F4/80**	AF700	1:100	BM8	123130, Biolegend	Rat IgG2a, κ
	780/60	Red A	**Ly6G/C**	APC eFluor 780	1:200	RB6-8C5	47-5931-82, Invitrogen	Rat IgG2b, κ
Yellow Green	586/15	YG C	**CD11c**	PE	1:200	N418	12-0114-83, eBiosciences	Armenian Hamster
	780/60	YG A	**CD45**	pecy7	1:800	30-F11	552848, BD Pharmingen	Rat IgG2b, κ
