# Supplementary material for: The temporal gene expression landscape of rhabdomyolysis-induced acute kidney injury reveals the timing of complement activation
Source: Commun Biol. 2025 Dec 31;9:171. doi: 10.1038/s42003-025-09449-y (PMC12877018; doi:10.1038/s42003-025-09449-y)
Supplement: Supplementary file 2 — Description of additional supplementary file [file 42003_2025_9449_MOESM2_ESM.pdf]

Description of additional supplementary file

File name: Supplementary Data

Description: Numerical raw data
