# Supplementary figures and images for: The temporal gene expression landscape of rhabdomyolysis-induced acute kidney injury reveals the timing of complement activation
Source: Commun Biol. 2025 Dec 31;9:171. doi: 10.1038/s42003-025-09449-y (PMC12877018; doi:10.1038/s42003-025-09449-y)

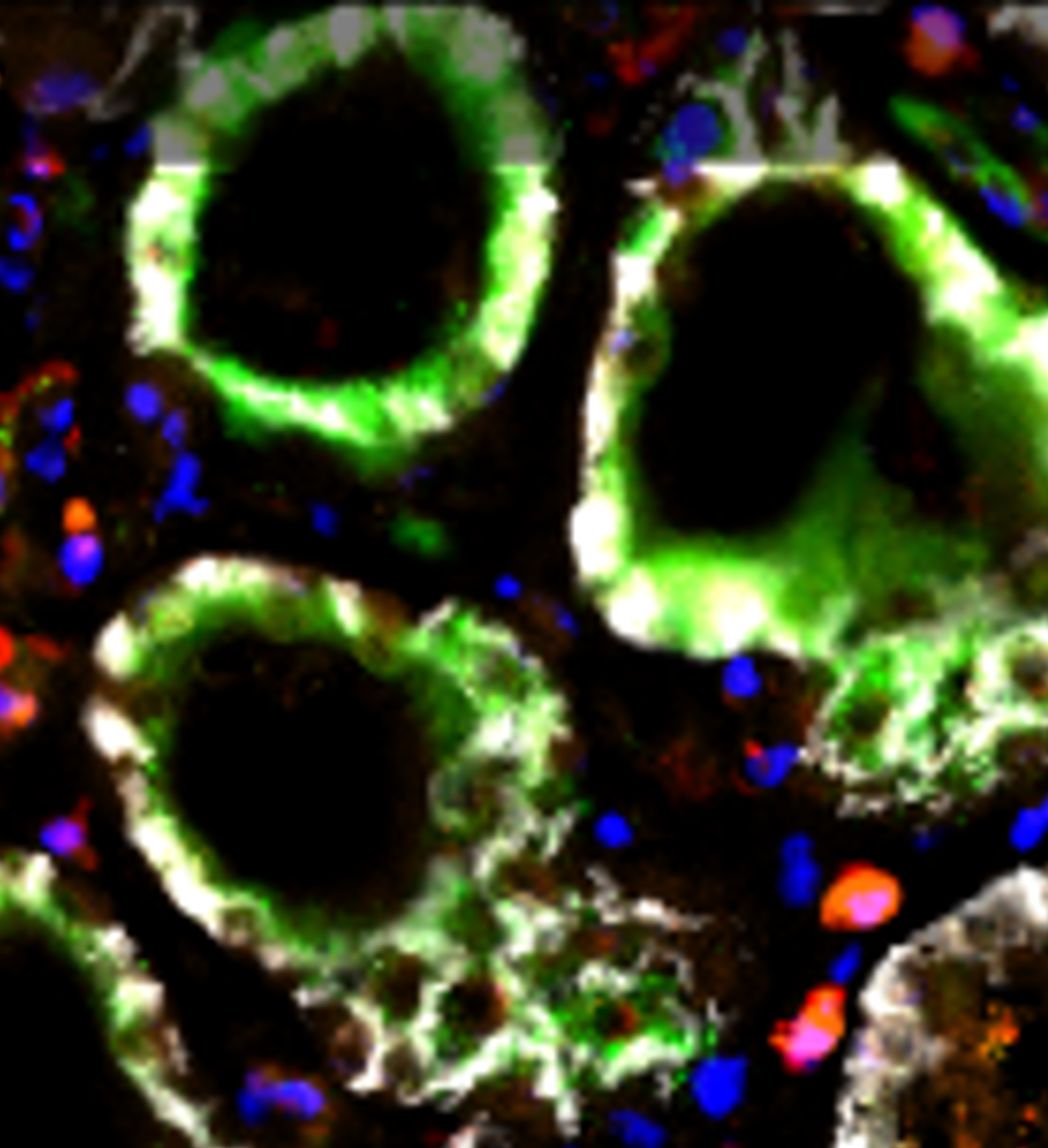

Supplement: Supplementary file 4 — Featured image [file 42003_2025_9449_MOESM4_ESM.tif]
